# Senataxin controls meiotic silencing through ATR activation and chromatin remodeling

**DOI:** 10.1038/celldisc.2015.25

**Published:** 2015-09-29

**Authors:** Abrey J Yeo, Olivier J Becherel, John E Luff, Mark E Graham, Derek Richard, Martin F Lavin

**Affiliations:** 1 The University of Queensland, UQ Centre for Clinical Research (UQCCR), Brisbane, QLD, Australia; 2 School of Medicine, The University of Queensland, Herston, Brisbane, QLD, Australia; 3 School of Chemistry and Molecular Biosciences, The University of Queensland, St Lucia, Brisbane, QLD, Australia; 4 Children’s Medical Research Institute, The University of Sydney, Westmead, NSW, Australia; 5 Cancer and Ageing Research Program, Faculty of Health, Queensland University of Technology, Brisbane, QLD, Australia

**Keywords:** senataxin, DNA damage repair, transcription, meiosis, chromatin remodeling

## Abstract

Senataxin, defective in ataxia oculomotor apraxia type 2, protects the genome by facilitating the resolution of RNA–DNA hybrids (R-loops) and other aspects of RNA processing. Disruption of this gene in mice causes failure of meiotic recombination and defective meiotic sex chromosome inactivation, leading to male infertility. Here we provide evidence that the disruption of *Setx* leads to reduced SUMOylation and disruption of protein localization across the XY body during meiosis. We demonstrate that senataxin and other DNA damage repair proteins, including ataxia telangiectasia and Rad3-related protein-interacting partner, are SUMOylated, and a marked downregulation of both ataxia telangiectasia and Rad3-related protein-interacting partner and TopBP1 leading to defective activation and signaling through ataxia telangiectasia and Rad3-related protein occurs in the absence of senataxin. Furthermore, chromodomain helicase DNA-binding protein 4, a component of the nucleosome remodeling and deacetylase chromatin remodeler that interacts with both ataxia telangiectasia and Rad3-related protein and senataxin was not recruited efficiently to the XY body, triggering altered histone acetylation and chromatin conformation in *Setx*^*−/−*^ pachytene-staged spermatocytes. These results demonstrate that senataxin has a critical role in ataxia telangiectasia and Rad3-related protein- and chromodomain helicase DNA-binding protein 4-mediated transcriptional silencing and chromatin remodeling during meiosis providing greater insight into its critical role in gene regulation to protect against neurodegeneration.

## Introduction

Mutations in senataxin give rise to the autosomal recessive cerebellar disorder, ataxia oculomotor apraxia type 2 (AOA2) [1, 2], and also to autosomal dominant juvenile-onset, amyotrophic lateral sclerosis 4 [3, 4]. It is still unclear how mutations in the *Setx* gene, located in close proximity, give rise to dominant and recessive disorders. However, there is evidence that posttranslational modifications of senataxin may help explain involvement in these different disease forms [5]. Senataxin is homologous to the yeast RNA–DNA helicase, Sen1, a component of the Nrd1 complex, involved in RNA polymerase II transcription termination and processing of noncoding nucleolar RNAs [6–8]. Similar to that reported for other proteins mutated in cerebellar ataxias, senataxin appears to have a role in protecting the genome against DNA damage [9–11]. Greater insight into its role in the response to DNA damage has been provided in recent studies. This protein was reported to interact with proteins involved in transcription and was shown to have a role in transcription termination, mRNA splicing efficiency and splice site selection [12]. Insight into its role in transcriptional termination was provided by the observation that senataxin resolves RNA–DNA hybrids (R-loops) and as a consequence allows Xrn2 (5′>3′ exonuclease) access at the 3′ cleavage poly (A) site, nascent transcript degradation and consequently polymerase II release and termination [13]. In yeast, Sen1 has also been shown to protect the genome from R-loop-mediated DNA damage [14]. The continuing presence of R-loops can have a negative impact on transcription elongation, leading to collisions with DNA replication forks and/or compromising genomic integrity by being a source of hypermutation or causing hyper-recombination [15]. More recently, senataxin has been localized to sites of collision between components of the replisome and the transcriptional apparatus [16]. By recognizing and resolving R-loops, it has an important role at the interface of transcription and the DNA damage response [16]. Evidence for this also exists in yeast where Sen1 has a key role in coordinating replication and transcription [17]. Senataxin has also been shown to suppress the anti-viral transcriptional response to control viral biogenesis [18]. It appears to do so by negatively regulating IRF3-dependent expression and promoting early termination of RNA polymerase II. At present, it remains unclear as to whether R-loops are responsible for the neurodegenerative changes that characterize AOA2. We previously reported the accumulation of R-loops in proliferating cells of *Setx* mutant mice treated with the topoisomerase inhibitor, topotecan hydrochloride, but failed to detect any R-loops in the brains of mice treated with the same agent [19].

Disruption of the *Setx* gene in mice failed to reveal any significant neurological abnormalities or any evidence of neurodegeneration [20]. However, male mutant mice were infertile and there was evidence of reduced fertility in females. Male germ cells proceeded normally from spermatogonia up to the pachytene stage of meiosis in *Setx*^*−/−*^, but failed to form mature spermatids [20]. Successful generation of programmed DNA double-strand breaks (DSB) and the initiation of DNA repair was observed in *Setx*^*−/−*^ spermatocytes, but RAD51 foci were abnormally retained in the pachytene stage and DNA DSBs persisted, revealing a defect in homologous recombination. In addition, absence of Mlh1 foci in *Setx*^*−/−*^ spermatocytes provided further evidence of failure to complete recombination. *Setx*^*−/−*^ spermatocytes also showed an accumulation of R-loops and underwent apoptosis. Furthermore, senataxin localized to the XY chromosomes at the pachytene stage and abnormal diffusion of DNA damage rep (DDR) proteins into the XY chromatin domain was observed, predicting that the XY chromosomes would fail to be transcriptionally silenced in *Setx*^*−/−*^ pachytene cells, which turned out to be the case [20]. These results pointed to a key role for senataxin in both meiotic homologous recombination and in meiotic sex chromosome inactivation (MSCI).

In this study, we used the *Setx* mutant mouse model to investigate the role of senataxin in meiotic transcriptional silencing. Loss of senataxin caused a marked decrease in protein SUMOylation across the XY body during the pachytene stage of meiosis. Senataxin itself was SUMOylated as well as several other proteins that include the ataxia telangiectasia and Rad3-related protein (ATR)-interacting partner, ATRIP, and in its absence, there was a marked downregulation and mislocalization of both ATRIP and TopBP1. Downregulation of ATRIP resulted from the failure of NIMA (never in mitosis gene a)-related kinase 1 (NEK1) to localize to the XY body and this led to defective ATR signaling. Chromodomain helicase DNA-binding protein 4 (CHD4), a key component of the nucleosome remodeling and deacetylase (NuRD) histone deacetylase chromatin remodeling complex, which interacts with both ATR and senataxin, was not efficiently recruited to the XY body, leading to persistent histone acetylation of the XY chromosomes and altered chromatin structure in *Setx*^*−/−*^ spermatocytes. These data provide further evidence of a critical role for senataxin in transcriptional silencing.

## Results

### Senataxin is SUMOylated on the XY body

**Figure 1:**
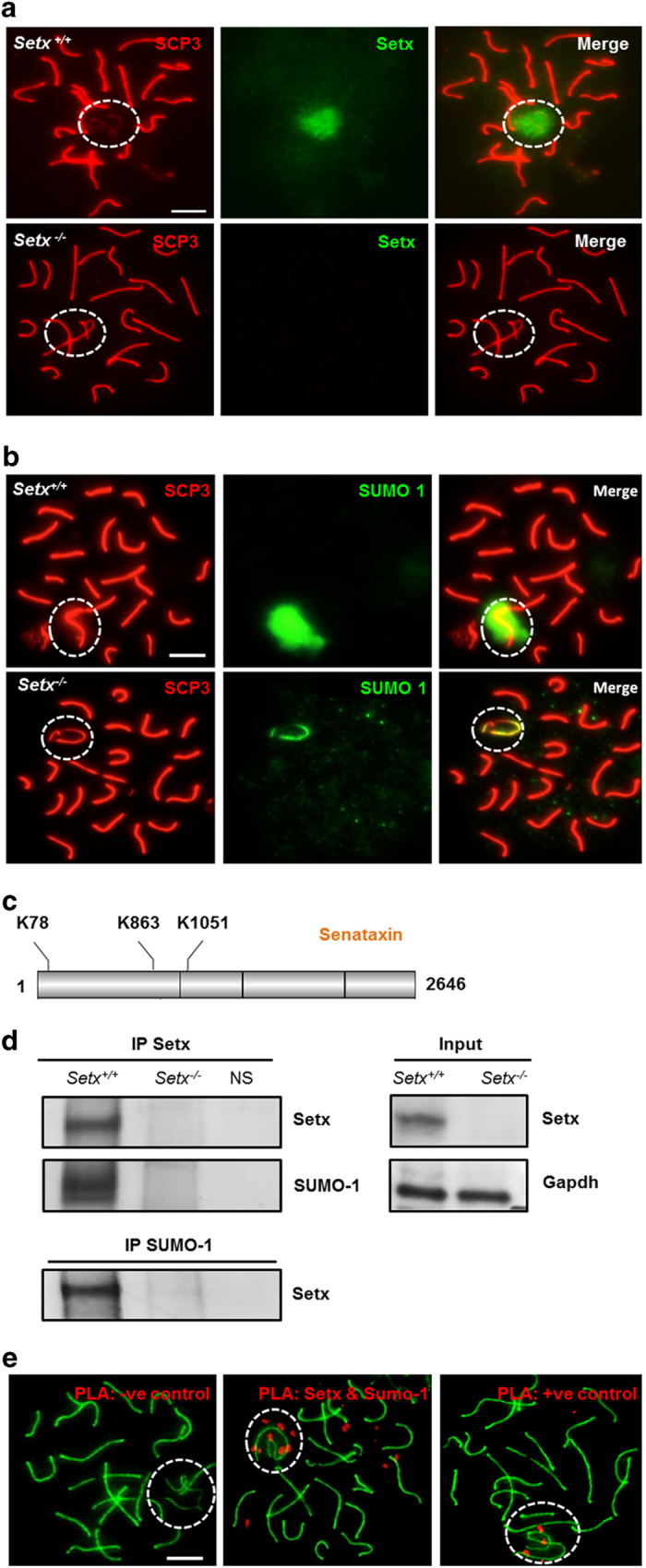
Senataxin is SUMOylated at the XY chromosomes in spermatocytes. (**a**) Immunostaining for senataxin revealed its localization to the XY chromosomes as a diffuse cloud during the pachytene stage in spermatocytes of *Setx*^*+/+*^ mice. Some background staining was also observed on the autosomes. As expected, no senataxin was detected in *Setx*^*−/−*^ spermatocytes. (**b**) Immunostaining for SUMO-1 in *Setx*^*+/+*^ and *Setx*^*−/−*^ spermatocytes revealed a diffuse staining over the XY chromosomes in *Setx*^*+/+*^ spermatocytes. In contrast, staining was largely restricted to the axes of the XY chromosomes in *Setx*^*−/−*^ spermatocytes. (**c**) GPS-SUMO, a bioinformatics tool, predicted three potential SUMOylation sites for senataxin. (**d**) Endogenous senataxin and SUMO-1 were co-immunoprecipitated and immunoblotted separately, showing SUMOylation of senataxin in *Setx*^*+/+*^ spermatocytes. A negative control for the immunoprecipitation (IP) using nonspecific (NS) immunoglobulin G (IgG) was also performed. As expected, input lanes show the presence of senataxin in *Setx*^*+/+*^ but not in *Setx*^*−/−*^ total cell extracts. Glyceraldehyde 3-phosphate dehydrogenase (GAPDH) was used as a loading control. (**e**) Through a proximity ligation assay (PLA), an interaction between senataxin and SUMO-1 was observed over the XY chromosomes as a concentration of red foci (middle panel). Some more scattered red foci were also observed over the autosomes. A negative control without a primary antibody (left panel) as well as a positive control (right panel) using antibodies against breast cancer type 1 susceptibility protein (BRCA1) and ataxia telangiectasia and Rad3-related protein (ATR), two proteins known to interact directly, was performed alongside. Scale bar, 20 μm. Synaptonemal complex protein 3 (SCP3), which indicates the axial elements of the synaptonemal complex. Dotted circle, XY chromosomes.

The various stages of meiosis can be differentiated by immunostaining for synaptonemal complex protein 3 (SCP3), a proteinaceous component of the axial element of the synaptonemal complex required for chromosomal synapsis [21]. We have previously shown that senataxin localized as a diffuse ‘cloud’ to the XY chromosomes in spermatocytes at the pachytene stage of male meiosis where it has an essential but undefined role in MSCI [20] (Figure 1a). Small ubiquitin-like modifier (SUMO) proteins have also been localized to the XY chromosomes and they have been implicated in the initiation of MSCI [20]. A yeast two-hydrid screen to detect SUMO-1- and SUMO-2/3-interacting motifs previously identified proteins involved in DNA repair and transcriptional repression [22]. and growing evidence implicated SUMO as an important posttranslational modification to modulate protein assemblies, activity and function [23]. Therefore, we investigated whether senataxin might be SUMOylated as part of its role in MSCI. Immunostaining for SUMO-1 of spermatocyte spreads revealed an intense, diffuse pattern of staining across the XY body in *Setx*^*+/+*^ spermatocytes (Figure 1b). On the other hand, when senataxin was disrupted, staining for SUMO-1 was significantly reduced and was confined to the unsynapsed region of the axial element of the XY chromosomes. A similar pattern of staining was observed with SUMO-2/3 (Supplementary Figure S1A). The early appearance of SUMO-1, which precedes the accumulation of γH2AX on the XY chromosomes, and the lack of SUMO-2/3 detection in zygotene spermatocytes, suggested a more specific role for SUMO-1 in the initiation of XY inactivation [24]. Therefore, SUMO-1 was chosen for further investigation. A bioinformatics tool, GPS-SUMO (http: //sumosp.biocuckoo.org), which predicts sites of SUMOylation and SUMO-interacting motifs based on consensus sequence predicted three sites of SUMOylation (Figure 1c) and three SUMO-interacting motifs in senataxin (Supplementary Figure S1B). All three potential SUMOylation sites are located in the N terminus of senataxin. Consistent with this prediction, Hecker *et al.* [22] previously identified senataxin as a SUMO-1/2-interacting protein using a yeast two-hybrid approach, and more recently an interaction between senataxin and Rrp45, a subunit of the exosome, was found to be dependent on the SUMOylation of senataxin [5]. Co-immunoprecipitation of senataxin and SUMO-1 was detected in cell extracts made from *Setx*^*+/+*^ testes, and as expected, no signal was detected in *Setx*^*−/−*^ extracts (Figure 1d). This was confirmed by the immunoprecipitation with an anti-SUMO-1 antibody (Figure 1d). Although immunoprecipitation confirmed the SUMOylation of senataxin, a proximity ligation assay (PLA) for both senataxin and SUMO-1 on *Setx*^*+/+*^ spermatocyte spreads was performed to determine the localization of the senataxin/SUMO-1 interaction at the pachytene stage. PLA allows the visualization of *in situ* endogenous protein–protein interactions by the appearance of fluorescent spots because of amplification and subsequent incorporation of fluorescent nucleotides on complementary strands that are conjugated to target antibodies [21]. The results provided further evidence that senataxin and SUMO-1 interacted with one another primarily on the XY chromosomes, as evidenced by the majority of the positive signals (red dots) focused over the XY body (Figure 1e). These results demonstrate that senataxin is SUMOylated and that it localizes primarily to the XY chromosomes.

### Defective SUMOylation of XY chromosome proteins in *Setx*^*−/−*^ pachytene cells

Given that the SUMOylation signal intensity was markedly decreased over the XY body and was mislocalized in the absence of senataxin, we predicted that other proteins localized to the XY body would depend on senataxin for their SUMOylation. To identify these SUMOylated proteins, we used immunoprecipitation with an anti-SUMO-1 antibody with extracts from *Setx*^*+/+*^ and *Setx*^*−/−*^ testes followed by sodium dodecyl sulfate-polyacrylamide gel electrophoresis (SDS-PAGE), and identification of Coomassie-stained bands by liquid chromatography mass spectrometry (LC-MS/MS). A stringent threshold for protein identification was set at *P*<0.01, the false discovery rate was set at 0.01% and a minimum of two unique peptides was used as an arbitrary threshold for confident protein identification. Several proteins were identified (Supplementary Table S1), which included several candidates related or potentially related to defective SUMOylation.

**Figure 2:**
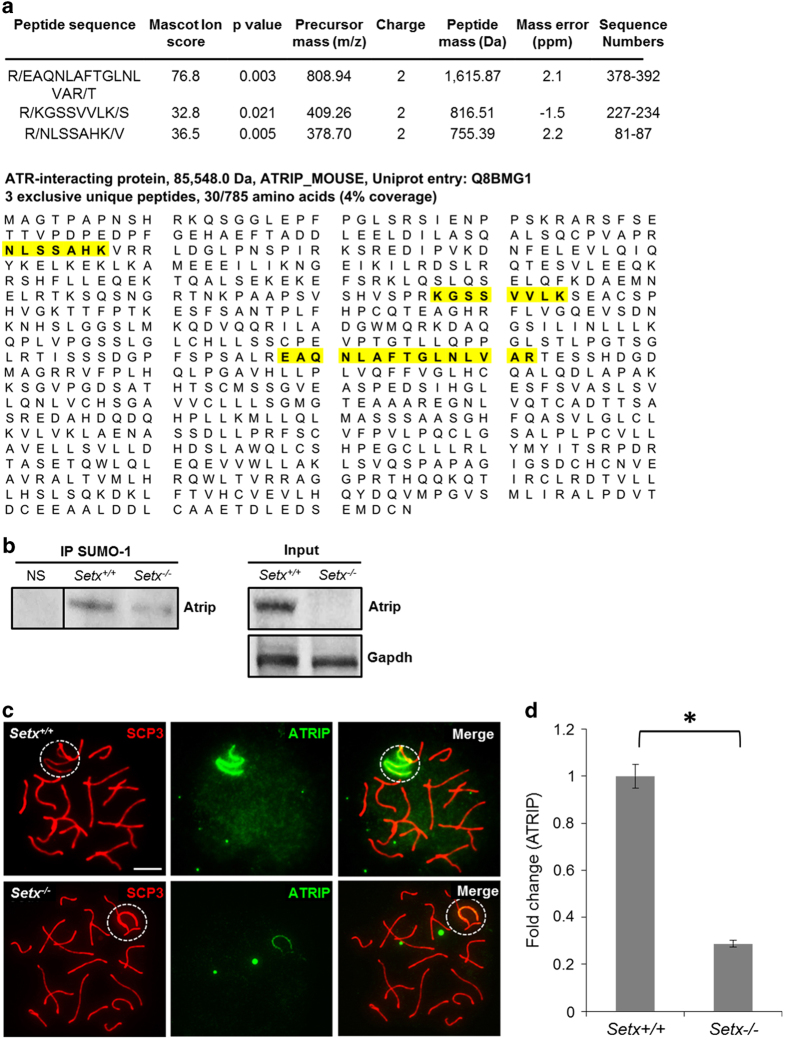
Ataxia telangiectasia and Rad3-related protein-interacting partner (ATRIP) protein levels are reduced in the absence of senataxin. (**a**) Liquid chromatography mass spectrometry (LC-MS/MS) on immunoprecipitation (IP) from *Setx*^*+/+*^ and *Setx*^*−/−*^ mouse testes using anti-SUMO-1 antibody revealed ATRIP as a candidate SUMOylated protein in *Setx*^*+/+*^ but not in *Setx*^*−/−*^ spermatocytes. Protein identification was visualized on the Scaffold Q+S application (Proteome Software, Portland, OR, USA). (**b**) IP from *Setx*^*+/+*^ and *Setx*^*−/−*^ testes extracts for SUMO-1 and immunoblotting for ATRIP confirmed the SUMOylation of ATRIP. A negative control for the IP using nonspecific (NS) immunoglobulin G (IgG) was also performed. Input lanes show the presence of ATRIP in *Setx*^*+/+*^ but not in *Setx*^*−/−*^ total cell extracts. Glyceraldehyde 3-phosphate dehydrogenase (GAPDH) was used as a loading control. (**c**) Immunostaining for ATRIP revealed a strong signal on the axial element of the XY chromosomes in *Setx*^*+/+*^ spermatocytes. On the other hand, decreased staining intensity for ATRIP was observed in *Setx*^*−/−*^ spermatocytes. Scale bar, 20 μm. SCP3, Synaptonemal complex protein 3. Dotted circle, XY chromosomes. (**d**) Quantitation of ATRIP fluorescence intensity staining (shown as fold change) in *Setx*^*+/+*^ and *Setx*^*−/−*^. Data are plotted as the mean±s.d. Statistical analysis was performed using the Student’s *t*-test, *P<0.05. Number of animals, *n*=3.

Among the SUMOylated proteins or those bound to SUMOylated proteins were those involved in chromatin remodeling (ATRX and RUVB2), in transcription regulation (DHX15 and YBOX3) and the regulatory partner of ATR, ATRIP, consistent with a role for SUMOylation in the DNA damage response and transcription. Data for ATRIP identification are presented in Figure 2a. We confirmed the SUMOylation of ATRIP in spermatocytes by carrying out immunoprecipitation with anti-SUMO-1 antibody and immunoblotting with anti-ATRIP antibody (Figure 2b). A reduction in ATRIP SUMOylation was observed in *Setx*^*−/−*^ spermatocytes as compared with *Setx*^*+/+*^, suggesting a lower level of ATRIP in *Setx*^*−/−*^. Indeed, this is in agreement with the difficulty of detecting endogenous levels of ATRIP in total cell extracts from *Setx*^*−/−*^ (Figure 2b). Furthermore, the decrease in ATRIP protein levels observed in *Setx*^*−/−*^ testes was not a defect in the expression of *ATRIP* (Supplementary Figure S3B), suggesting that senataxin does not directly affect the transcriptional regulation of *ATRIP*.

The reduction of ATRIP, and consequently SUMOylated ATRIP, in *Setx*^*−/−*^ spermatocytes and the importance of ATRIP in maintaining the stability and activity of ATR prompted us to investigate the localization of ATRIP in spermatocytes from these mice. A strong signal for ATRIP that was restricted largely to the axial element of the XY chromosomes was observed in *Setx*^*+/+*^ pachytene spermatocytes (Figure 2c). Additionally, some background staining for ATRIP was observed on the autosomes. Although the localization of ATRIP in *Setx*^*−/−*^ spermatocytes was similar to that observed in *Setx*^*+/+*^ spermatocytes, the signal was markedly reduced. Thus, a reduction in ATRIP protein levels and/or defective recruitment to the XY chromosomes appears to occur in *Setx*^*−/−*^ pachytene-staged spermatocytes. Quantitation of ATRIP fluorescence intensity staining on the XY chromosomes revealed an ~3.3-fold reduction in *Setx*^*−/−*^ compared with *Setx*^*+/+*^ (Figure 2d).

### Activation of ATR and downstream signaling is defective in *Setx*^*−/−*^ spermatocytes

**Figure 3:**
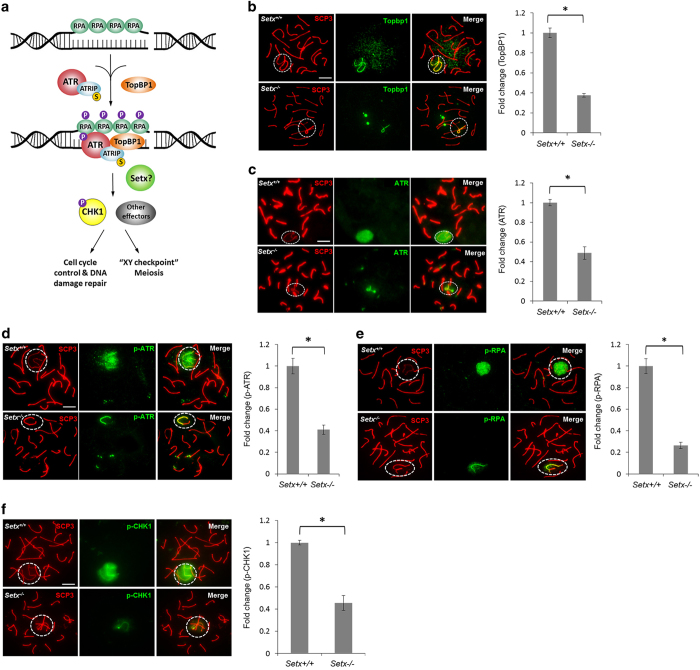
Ataxia telangiectasia and Rad3-related protein (ATR) activity and signaling is defective in the absence of senataxin. (**a**) A schematic illustration of the ATR signaling pathway and the proteins investigated. (**b**) TopBP1 was observed to localize predominantly to the axial element of the XY chromosomes in *Setx*^*+/+*^ spermatocytes. In contrast, decreased staining intensity for TopBP1 was observed in *Setx*^*−/−*^ spermatocytes. Quantitation of TopBP1 fluorescence intensity staining (shown as fold change) in *Setx*^*+/+*^ and *Setx*^*−/−*^. (**c**) ATR localized as a cloud in *Setx*^*+/+*^ spermatocytes, whereas in *Setx*^*−/−*^, ATR was confined to the axial element of the XY chromosomes. Quantitation of ATR fluorescence intensity staining (shown as fold change) in *Setx*^*+/+*^ and *Setx*^*−/−*^. (**d**) Immunostaining for phospho-S428 ATR (p-ATR(S428) showed a cloud over the XY chromosomes in *Setx*^*+/+*^ spermatocytes as expected, whereas phospho-S428 ATR (p-ATR) was retained on the axial element of the XY chromosomes in *Setx*^*−/−*^ spermatocytes. Quantitation of p-ATR fluorescence intensity staining (shown as fold change) in *Setx*^*+/+*^ and *Setx*^*−/−*^. (**e**) Immunostaining for p-RPA(S33) showed a diffuse staining pattern over the XY body in *Setx*^*+/+*^ spermatocytes as expected, whereas it was largely retained on the axial element of the XY chromosomes in *Setx*^*−/−*^ spermatocytes. Quantitation of p-RPA fluorescence intensity staining (shown as fold change) in *Setx*^*+/+*^ and *Setx*^*−/−*^. (**f**) Localization of phospho-S317 CHK1(S317) (p-CHK1(S317)) to XY chromatin in *Setx*^*+/+*^. Reduced levels of p-CHK1(S317) localized mainly to the axial element of the XY chromosomes in *Setx*^*−/−*^ spermatocytes. Quantitation of p-CHK1 fluorescence intensity staining (shown as fold change) in *Setx*^*+/+*^ and *Setx*^*−/−*^. Scale bar, 20 μm. Synaptonemal complex protein 3 (SCP3). Dotted circle, XY chromosomes. All data were plotted as the mean±s.d. Statistical analysis was performed using the Student’s *t*-test, *P<0.05. Number of animals, *n*=3.

ATR has also been shown to have a key role in MSCI where it is required for the phosphorylation of H2AX [25]. Briefly, ATR is a kinase that is activated upon recognition of DNA damage, which includes single-stranded DNA breaks [26]. Single-stranded DNA is first detected and coated by replication protein A (RPA), and subsequently the ATR-ATRIP complex is recruited to sites of DNA damage and activated by TopBP1, leading to the activation/phosphorylation of its downstream substrates, which include RPA and checkpoint kinase 1 (CHK1) (Figure 3a) [27, 28]. The initiation of MSCI begins with the recruitment of breast cancer type 1 susceptibility protein (BRCA1) to the axes of the XY chromosomes, which then promotes ATR recruitment [25]. As shown in Supplementary Figure S2, normal recruitment of BRCA1 was observed in both *Setx*^*+/+*^ and *Setx*^*−/−*^ mice, indicating that the initiation of MSCI occurs typically in these mice. This then suggested that the defect in MSCI in *Setx*^*−/−*^ spermatocytes lies downstream of this initial step. As TopBP1 acts as a general activator of ATR [22] and this activation is mediated through ATRIP, we predicted that there might also be a defect in ATR activation and downstream signaling in *Setx*^*−/−*^ spermatocytes. Immunostaining on spermatocytes of *Setx* mice showed that TopBP1 localized predominantly on the axial elements of the XY chromosomes in *Setx*^*+/+*^ spermatocytes as expected, whereas a much fainter signal was observed in *Setx*^*−/−*^ spermatocytes, despite a similar localization to that observed in *Setx*^*+/+*^ (Figure 3b). Quantitation of fluorescence intensity of TopBP1 also showed an ~2.6-fold reduction in *Setx*^*−/−*^ as compared with *Setx*^*+/+*^ (Figure 3b). These results suggested a potential defect at the level of ATR function and downstream signaling in the absence of senataxin. In addition to coordinating DDR, ATR is required for the phosphorylation of histone H2AX on the XY chromosomes during MSCI [25]. Although the exact purpose of this phosphorylation remains unclear, it has been hypothesized that it is necessary for initiating chromatin condensation and repression of transcription [29]. We observed that ATR formed a diffuse cloud over the XY body in *Setx*^*+/+*^ spermatocytes as reported previously [30], whereas it was localized largely on the axial element of the XY chromosomes in *Setx*^*−/−*^ spermatocytes, failing to diffuse out into XY chromatin (Figure 3c). Quantitation of fluorescence intensity of ATR also showed an ~2-fold reduction in *Setx*^*−/−*^ as compared with *Setx*^*+/+*^ (Figure 3c). To determine whether the altered localization of ATR might reflect a difference in its activity, immunostaining against an active form of ATR, phospho-S428 ATR (p-ATR) was performed. Results showed a similar staining pattern to that observed for ATR, with staining being confined to the unsynapsed region of the axial elements of the XY chromosomes (Figure 3d). Quantitation of fluorescence intensity of p-ATR also showed an ~2.4-fold reduction in *Setx*^*−/−*^ as compared with *Setx*^*+/+*^ (Figure 3d). These data point to a defect in the diffusion of p-ATR, which could lead to alterations in the downstream DDR signaling that occurs during MSCI. To examine this possibility, we next investigated ATR signaling in the absence of senataxin by immunostaining for two ATR substrates, RPA and CHK1 [31, 32]. Phospho-S33 RPA (p-RPA) was observed to form an intense diffuse cloud over the XY chromosomes in *Setx*^*+/+*^ spermatocytes, whereas p-RPA was significantly reduced in intensity and localized mainly on the axial element of the XY chromosomes in *Setx*^*−/−*^ spermatocytes (Figure 3e). Quantitation of fluorescence intensity of p-RPA also showed an ~2.2-fold reduction in *Setx*^*−/−*^ as compared with *Setx*^*+/+*^ (Figure 3e). CHK1, a kinase required for cell cycle checkpoint activation and a well-documented downstream target of ATR for phosphorylation [33, 34], was next assessed. The ATR-phosphorylated form of CHK1 phospho-S317 CHK1 (p-CHK1) formed a diffuse cloud over the XY chromosomes in *Setx*^*+/+*^ spermatocytes, whereas a reduced signal that was largely retained on the axial element was observed in *Setx*^*−/−*^ spermatocytes (Figure 3f). Quantitation of fluorescence intensity of p-CHK1 also showed an ~3.8-fold reduction in *Setx*^*−/−*^ as compared with *Setx*^*+/+*^ (Figure 3f). Both p-RPA and p-CHK1 exhibited similar staining patterns as those of ATR and p-ATR in *Setx*^*+/+*^ and *Setx*^*−/−*^ spermatocytes. Immunoblotting was carried to determine the levels of the proteins involved in ATR signaling. Both ATRIP and TopBP1 protein levels were markedly reduced in *Setx*^*−/−*^, in agreement with the immunofluorescence data (Supplementary Figure S3A). However, similar levels of *TopBP1* and *ATRIP* mRNAs were observed between *Setx*^*+/+*^ and *Setx*^*−/−*^, suggesting that senataxin does not directly regulate the expression of these genes at the transcriptional level (Supplementary Figure S3B). Although ATR and CHK1 were present in normal levels, there was evidence that ATR activation was lower in *Setx*^*−/−*^ as observed with decreased p-ATR protein levels (Supplementary Figure S3A). Taken together, these data support a defect in ATR activity and downstream signaling in *Setx*^*−/−*^ as this activity is confined to the axial elements of the XY chromosomes, which could account for the failure of MSCI in these mice.

**Figure 4:**
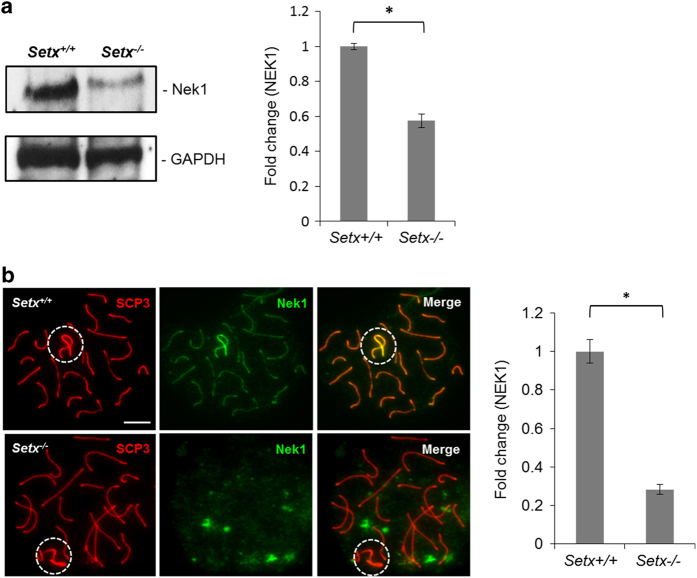
NNIMA (never in mitosis gene a)-related kinase 1 (NEK1) localization and protein levels are disrupted in the absence of senataxin. (**a**) Immunoblotting from testes extracts revealed a decrease in NEK1 protein levels in *Setx*^*−/−*^ as compared with *Setx*^*+/+*^ spermatocytes. α-Tubulin was used as a loading control. Quantitation of NEK1 band intensity staining (shown as fold change) in *Setx*^*+/+*^ and *Setx*^*−/−*^. (**b**) NEK1 was observed to localize specifically to the axial element of the autosomes with a stronger intensity on the XY chromosomes in *Setx*^*+/+*^ spermatocytes, whereas NEK1 in *Setx*^*−/−*^ spermatocytes was not associated with the XY chromosomes but was scattered throughout the nucleus. Quantitation of NEK1 fluorescence intensity staining (shown as fold change) in *Setx*^*+/+*^ and *Setx*^*−/−*^. Scale bar, 20 μm. Dotted circle, XY chromosomes. All data were plotted as the mean±s.d. Statistical analysis was performed using the Student’s *t*-test, *P<0.05. Number of animals, *n*=3.

A recent report showed that the kinase activity of NEK1 is critical for initiating the ATR response following DNA damage [35]. Cells lacking NEK1 failed to support ATR autophosphorylation, did not efficiently phosphorylate ATR downstream substrates and exhibited significantly reduced levels of ATRIP [35]. Thus, it is possible that in *Setx*^*−/−*^ cells, a defect in NEK1 may be present, which could in turn account for the defect in ATR activity observed here, and consequently a failure in MSCI. Immunoblotting for NEK1 protein from total cell extracts from testes of *Setx*^*+/+*^ and *Setx*^*−/−*^ mice revealed an ~1.7 fold decrease in NEK1 in *Setx*^*−/−*^ (Figure 4a), despite similar gene expression levels (Supplementary Figure S3B). Immunostaining showed that NEK1 localized to the axial element of the autosomes, with a stronger signal on the XY chromosomes in *Setx*^*+/+*^ spermatocytes (Figure 4b). In contrast, NEK1 localized neither to the autosomes nor the XY chromosomes in *Setx*^*−/−*^ spermatocytes, but rather formed a diffuse staining pattern throughout the nucleus. Quantitation of fluorescence intensity of NEK1 also showed an ~3.6-fold reduction in *Setx*^*−/−*^ as compared with *Setx*^*+/+*^ (Figure 4b). The decreased protein level of NEK1 as well as its mislocalization in *Setx*^*−/−*^ spermatocytes suggests that senataxin may modulate the expression indirectly and/or localization of NEK1, and that NEK1 disruption may in turn account for the destabilization of ATRIP and the defect in ATR signaling observed in *Setx*^*−/−*^ spermatocytes.

### Lack of CHD4 recruitment to the XY body in the absence of senataxin

**Figure 5:**
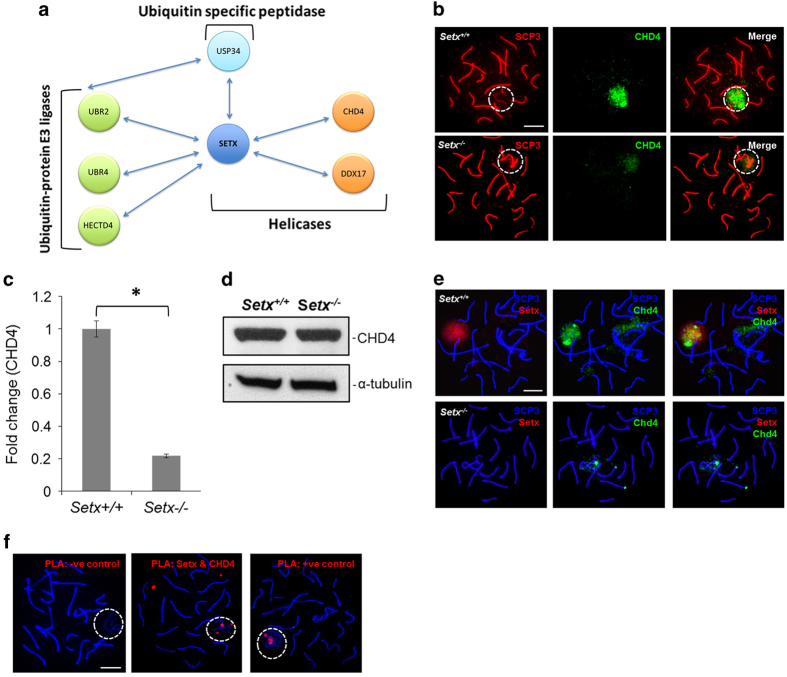
Senataxin interacts with and affects chromodomain helicase binding DNA protein 4 (CHD4) localization. (**a**) Pathway analysis of the senataxin connectivity map was performed using data from PCViz Pathway Commons Network Visualizer (http://www.pathwaycommons.org); this demonstrated that senataxin was coregulated with three ubiquitin ligases and an ubiquitin peptidase, along with two other helicases. (**b**) Immunostaining for CHD4 revealed a strong signal on the XY chromosomes in *Setx*^*+/+*^ spermatocytes, whereas decreased levels of CHD4 were observed in *Setx*^*−/−*^ spermatocytes. (**c**) Quantitation of CHD4 fluorescence intensity staining (shown as fold change) in *Setx*^*+/+*^ and *Setx*^*−/−*^. All data were plotted as the mean±s.d. Statistical analysis was performed using the Student’s *t*-test, *P<0.05. Number of animals, *n*=3. (**d**) Immunoblotting from testes extracts for CHD4 revealed similar levels of this protein in both *Setx*^*−/−*^ and *Setx*^*+/+*^ mice. (**e**) Co-immunofluorescence of senataxin and CHD4 revealed overlapping staining over the XY domain in *Setx*^*+/+*^ spermatocytes. (**f**) Proximity ligation assay (PLA) between senataxin and CHD4 revealed an interaction between these proteins at the XY chromosomes in spermatocytes of *Setx*^*+/+*^ mice (middle panel). A negative control without primary antibody (left panel) as well as a positive control (right panel) using antibodies against breast cancer type 1 susceptibility protein (BRCA1) and ATR, two proteins known to interact directly, was performed alongside. Scale bar, 20 μm. Dotted circle, XY chromosomes.

We have previously shown that in the absence of senataxin, failure of MSCI is associated with differential chromatin conformation as observed by defective ubiquitination of histone H2A [20]. The abnormal ATR activation and signaling observed in *Setx*^*−/−*^ spermatocytes and the alterations in X- and Y-linked gene expression in *Setx*^*−/−*^ spermatocytes observed previously [20] could also result from defective chromatin remodeling. Relevant to this, the CHD4, a member of the NuRD complex [36], was shown previously to interact with senataxin in HeLa cells [16]. Gene expression connectivity mapping provided additional evidence that senataxin and CHD4 are interacting partners (Figure 5a). Connectivity mapping is an advanced bioinformatics technique that is used to establish the connections among different biological states via their expression profiles/signatures [37]. In addition to identifying CHD4 as a senataxin-interacting protein, other proteins implicated in transcription control were also identified (Figure 5a). CHD4 has also been reported to interact with ATR [38], suggesting a link between its role in mediating checkpoints induced by DNA damage and chromatin remodeling by deacetylation [39]. Immunostaining for CHD4 revealed a similar pattern of localization to the XY chromosomes in *Setx*^*+/+*^ and *Setx*^*−/−*^ spermatocytes (Figure 5b). Quantitation of CHD4 fluorescence intensity staining on the XY chromosomes revealed an ~5-fold reduction in *Setx*^*−/−*^ compared with *Setx*^*+/+*^ (Figure 5c). This was not explained by reduced protein, as similar levels of CHD4 protein were observed in both *Setx*^*+/+*^ and *Setx*^*−/−*^ testes extracts (Figure 5d), indicating that the faint CHD4 staining in *Setx*^*−/−*^ spermatocytes most likely resulted from a defect in its recruitment to the XY chromosomes. Although CHD4 was shown to interact with senataxin by co-immunoprecipitation [16] and predicted from connectivity mapping in somatic cells, it was important to determine whether senataxin and CHD4 interacted *in vivo* in spermatocytes. Immunostaining for senataxin and CHD4 revealed colocalization on the XY chromosomes (Figure 5e). Using PLA, we revealed an *in situ* interaction between senataxin and CHD4 at the XY chromosomes in *Setx*^*+/+*^ spermatocytes (Figure 5f), thus providing further evidence for the *in vivo* interaction between these two proteins. This suggests that senataxin and ATR are involved in the recruitment of CHD4 to the XY body where they function together in chromatin remodeling required for XY silencing.

### Persistence of histone acetylation on the XY chromosomes in *Setx*^*−/−*^ spermatocytes

**Figure 6:**
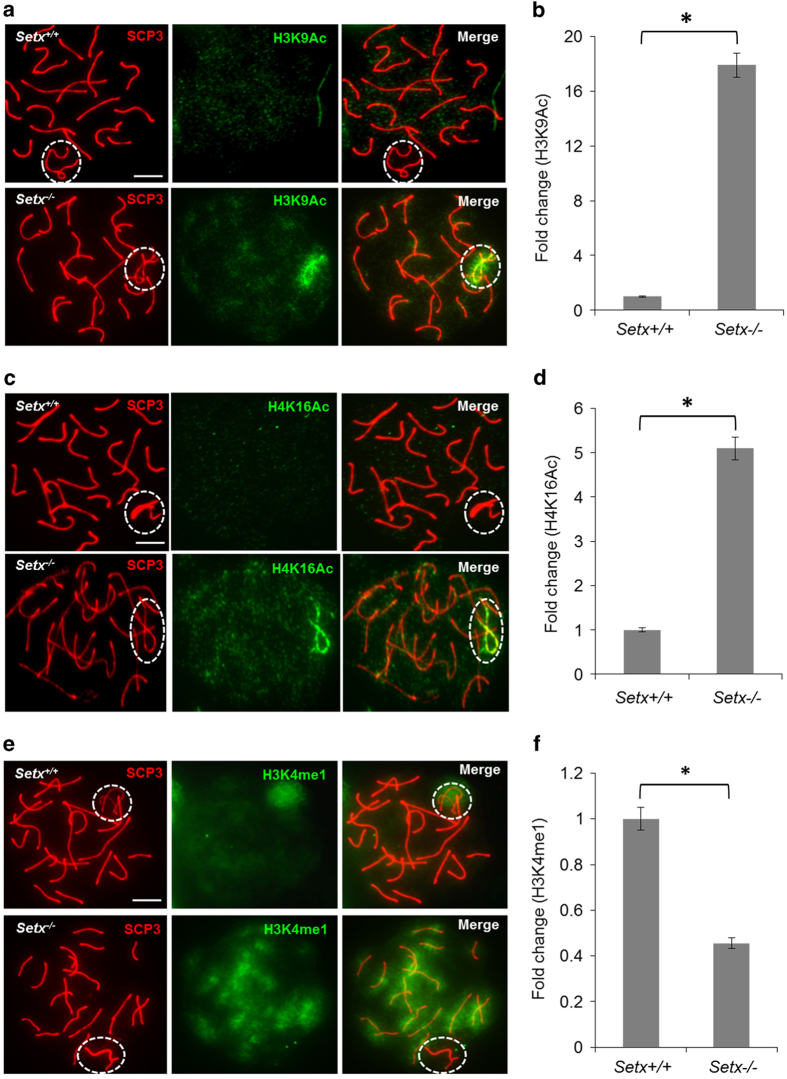
Differential XY chromatin topology contributes to the failure of meiotic sex chromosome inactivation (MSCI) in *Setx*^*−/−*^ spermatocytes. (**a**) Immunostaining for the epigenetic marker of acetylation, histone H3 acetyl K9 (H3K9Ac), revealed hyperacetylation of the XY chromosomes only in *Setx*^*−/−*^ spermatocytes. No signals for these histone modifications were observed over the XY domain of *Setx*^*+/+*^ spermatocytes as expected. (**b**) Quantitation of H3K9Ac fluorescence intensity staining (shown as fold change) in *Setx*^*+/+*^ and *Setx*^*−/−*^. (**c**) Histone H4 at lysine 16 (H4K16Ac) staining revealed similar hyperacetylation of the XY chromosomes only in *Setx*^*−/−*^ spermatocytes. (**d**) Quantitation of H4K16Ac fluorescence intensity staining (shown as fold change) in *Setx*^*+/+*^ and *Setx*^*−/−*^. (**e**) Immunostaining for the histone methylation mark, H3K4me1, revealed specific staining over the XY chromosomes in *Setx*^*+/+*^, compatible with transcriptional silencing, but not in *Setx*^*−/−*^ spermatocytes. (**f**) Quantitation of H3K4me1 fluorescence intensity staining (shown as fold change) in *Setx*^*+/+*^ and *Setx*^*−/−*^. Scale bar, 20 μm. Dotted circle, XY chromosomes. All data were plotted as the mean±s.d. Statistical analysis was performed using the Student’s *t*-test, *P<0.05. Number of animals, *n*=3.

CHD4 functions as part of the NuRD complex to control the programming of cell states during development, triggering chromatin conformation changes by posttranslational modifications, and consequently changes in transcriptional activity [38–40]. This complex is capable of both opening and closing chromatin, with a recent report demonstrating that CHD4 is the most frequent participant in chromatin closing events [41]. A defect in the recruitment of CHD4 to the XY body is predicted to result in more open chromatin, compatible with the presence of active RNA polymerase II transcription and MSCI failure in *Setx*^*−/−*^ spermatocytes [20]. To address this further, we determined the status of histone posttranslational modifications such as histone H3 acetyl K9 (H3K9Ac), a histone modification associated with transcription [42]. As expected, this modification was only observed on the autosomes and not on the XY chromosomes in *Setx*^*+/+*^ pachytene-staged spermatocytes, consistent with the onset of MSCI, whereas H3K9Ac strongly lit up the XY chromosomes in *Setx*^*−/−*^ spermatocytes (Figure 6a). Quantitation of H3K9Ac fluorescence intensity staining on the XY chromosomes revealed an ~17-fold increase in *Setx*^*−/−*^ compared with *Setx*^*+/+*^ (Figure 6b). Further evidence of open chromatin on the XY chromosomes was provided by analyzing the spread of histone H4 at lysine 16 (H4K16Ac), another histone modification associated with transcriptionally active chromatin [42]. Similarly, H4K16Ac signal was not on the XY chromosomes of *Setx*^*+/+*^ spermatocytes, whereas a strong signal was observed on the XY chromosomes of *Setx*^*−/−*^ spermatocytes with additional background signals observed on the autosomes (Figure 6c). Quantitation of H4K16Ac fluorescence intensity staining on the XY chromosomes revealed an ~5-fold increase in *Setx*^*−/−*^ compared with *Setx*^*+/+*^ (Figure 6d). Contrary to acetylation, histone methylation is an indicator of transcriptional repression [42]. To confirm the major difference in chromatin conformation in *Setx*^*−/−*^ spermatocytes, H3K4me1, an epigenetic marker associated with transcription repression [42], was also examined. Immunostaining against this marker revealed a strong signal over the XY chromosomes in *Setx*^*+/+*^ spermatocytes, consistent with transcriptional repression and silencing (Figure 6e). In contrast, signal for H3K4me1 was not observed on the XY chromosomes of *Setx*^*−/−*^ spermatocytes but enhanced signals for this marker was observed on the autosomes, suggesting a more general genome-wide defect in chromatin topology in *Setx*^*−/−*^ mice (Figure 6e). Quantitation of H3K4me1 fluorescence intensity staining on the XY chromosomes revealed an ~2.5-fold reduction in *Setx*^*−/−*^ compared with *Setx*^*+/ +*^(Figure 6f). Altogether, the pattern of histone acetylation and methylation points to abnormalities in chromatin conformation leading to gene activation on the XY body, which is consistent with the MSCI failure previously observed in *Setx*^*−/−*^ pachytene-staged spermatocytes.

### Concerted action of senataxin and CHD4 in regulating XY silencing

**Figure 7:**
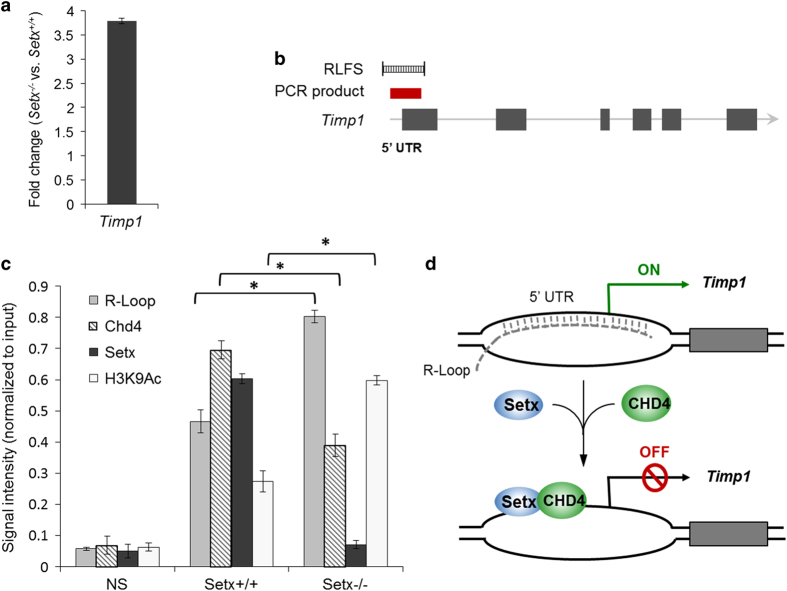
Coordinated action of senataxin and chromodomain helicase DNA-binding protein 4 (CHD4) in meiotic silencing. (**a**) Increased levels of expression of *Timp1* in *Setx*^*−/−*^ germ cells. (**b**) Diagram depicting the *Timp1* gene structure, the predicted R-loop forming site (RLFS; black bars) located upstream and in the 5′-untranslated region (5′-UTR) region of this gene and the corresponding polymerase chain reaction (PCR) product (red bar) used in chromatin immunoprecipitation (ChIP) assays. (**c**) DNA:RNA immunoprecipitation (DRIP), SETX, CHD4 and histone H3 Lys 9Ac (H3K9Ac) ChIP in *Setx*^*+/+*^ and *Setx*^*−/−*^ germ cells revealed the accumulation of R-loops, the reduced binding of CHD4 and the increased of histone H3 Lys9 (H3K9) acetylation in *Setx*^*−/−*^ cells. Similar levels of senataxin and CHD4 binding was observed in *Setx*^*+/+*^ germ cells, whereas an ~2-fold reduction in CHD4 recruitment and only background levels of binding for senataxin similar to those obtained with negative control (nonspecific (NS)) immunoglobulin G (IgG) were observed in the knockout. All data were plotted as the mean±s.d. Statistical analysis was performed using the Student’s *t*-test, *P<0.05. Number of animals, *n*=3. (**d**). Model depicting the coordinated action of senataxin and CHD4 in silencing *Timp1* during meiosis. Briefly, under normal conditions R-loops form at the GC-rich region upstream at the 5′-UTR of the *Timp1* gene. Recruitment of senataxin and CHD4 occurs and leads to R-loop resolution, histone deacetylation and silencing of *Timp1* during meiosis.

Based on the localization and *in situ* interaction of senataxin and CHD4 on the XY chromosomes and the aberrant histone acetylation marks observed in *Setx*^*−/−*^ pachytene spermatocytes, we decided to investigate the relationship between R-loop formation, senataxin and CHD4 in modulating XY silencing. GC skew is a common characteristic of human CpG islands and R-loops have been shown to interfere with CpG island methylation at promoter sites, which can then lead to silencing defects [43]. To gain further mechanistic insight into MSCI, we first determined whether R-loop formation may modulate the expression of XY genes by using microarray analysis, and subsequently carrying out *in silico* analysis for R-loops formation sites (RLFS) on aberrantly expressed XY genes in *Setx*^*−/−*^. The mouse *Timp1* gene, which displayed increased expression in *Setx*^*−/−*^ (Figure 7a), was found to contain a predicted RLFS upstream of its 5'-untranslated region (UTR) (Figure 7b). We next performed DNA:RNA immunoprecipitation (DRIP) using the S9.6 antibody, an antibody specific for R-loops (Supplementary Figure S4), to confirm the presence of these structures at this locus. As shown in Figure 7c, a higher level of R-loops (~2-fold increase) was observed at the *Timp1* locus in *Setx*^*−/−*^ as compared with *Setx*^*+/+*^. Chromatin immunoprecipitation (ChIP) with senataxin also revealed its binding to this locus in *Setx*^*+/+*^. As expected, only background levels of binding were observed in *Setx*^*−/−*^ (Figure 7c). These data are in agreement with the role of senataxin in resolving R-loops [13]. Interestingly, the 5′-UTR region of *Timp1* has been found to be methylated in germ cells under normal conditions (Supplementary Figure S5), suggesting that the absence of senataxin may lead to an increase in R-loop formation at this locus, which may prevent the methylation of this GC-rich region in *Setx*^*−/−*^.

Gene regulation is primarily modulated by histone methylation and/or acetylation [44, 45]. Given the *in situ* interaction between senataxin and CHD4 on the XY chromosomes, and that CHD4 is a core component of the histone deacetylase NuRD complex [46], we next performed ChIP to investigate the recruitment of CHD4 to the *Timp1* locus and the histone acetylation status of this region. As shown in Figure 7c, CHD4 bound to this region to a similar extent as senataxin in *Setx*^*+/+*^, whereas an ~50% reduction in CHD4 binding was observed in *Setx*^*−/−*^. Additionally, reduced CHD4 binding to this locus was inversely correlated with a twofold increase in histone H3 Lys9 (H3K9) acetylation, which is in agreement with previous work demonstrating a role for CHD4 in modulating the modification of this site [46].

Altogether, these data indicate that (i) R-loops form at the 5'-UTR region of the *Timp1* locus under normal conditions, (ii) R-loops accumulate to a greater extent at this site in the absence of senataxin and (iii) the lack of senataxin hinders the recruitment of CHD4, which correlates with higher levels of histone acetylation at this locus and consequently increased *Timp1* expression. Thus, we propose a mechanism in which under normal conditions, the concomitant binding of senataxin and CHD4 to *Timp1* 5'-UTR leads to the silencing of *Timp1* through the resolution of R-loops and the deacetylation of H3K9 by the CHD4 (Figure 7d). In contrast, R-loops accumulate at the 5'-UTR in the absence of senataxin, preventing methylation of the region, and as CHD4 is not efficiently recruited, H3K9 acetylation is not removed, thus leading to the aberrant expression of *Timp1* during MSCI in *Setx*^*−/−*^.

## Discussion

We have previously shown that the disruption of *Setx* in mice leads to a defect in meiotic recombination and a failure in MSCI [20]. In this report, we have provided greater mechanistic insight into the role of senataxin in MSCI. Recent data suggest that meiotic silencing involves a complex interplay between ATR and other silencing effectors [47]. In that study, ATR was shown to have multiple roles in silencing, including the regulation of HORMA (Hop1, Rev7, Mad2) domain protein HORMAD1/2 phosphorylation and localization of ATRIP and TopBP1 to unsynapsed axes of the XY chromosomes. ATR subsequently transduces signaling at unsynapsed axes to the surrounding chromatin with the assistance of MDC1 and phosphorylates H2AX (γH2AX), the epigenetic event leading to gene inactivation. We have previously shown that in the absence of senataxin, while ATR localizes to the axial element of XY chromosomes, it does not diffuse out into the surrounding chromatin [20]. Nevertheless, under these conditions, phosphorylation of H2AX (across the XY body) appeared to be normal, suggesting that since ATR is responsible for this phosphorylation, it was not required to localize to the surrounding chromatin to achieve this or that perhaps another PIKK was involved in this phosphorylation. Royo *et al.* [47] envisaged that when ATR translocates into chromatin loops of unsynapsed regions, it leads to the induction of repressive posttranslational modifications, such as γH2AX, and causes irreversible gene silencing over several megabases. Our results suggest that phosphorylation of H2AX is not the rate-determining step in gene silencing and that other modifications to chromatin are also required.

As part of the response to DNA damage, ATRIP forms a stoichiometric complex with ATR and by binding directly to RPA, enables the accumulation of ATR at the sites of DNA damage [48]. Taken together with the involvement of a number of other regulatory factors, this leads to ATR activation to coordinate cell cycle checkpoints, replication fork stability and replication restart [49]. Not surprisingly, the mechanism of ATR activation is conserved in meiosis where ATRIP localizes with ATR, TopBP1 and RPA at unsynapsed regions of meiotic chromosomes [50]. A recent report revealed that ATRIP is SUMOylated in response to agents that disrupt DNA replication and that ATRIP mutants lacking SUMOylation sites failed to localize to DNA damage sites and did not support efficient ATR activation [51, 52]. In promoting ATR activation, SUMOylated ATRIP interacts with multiple proteins including ATR, TopBP1, RPA and the MRN complex not only through direct interaction but also through interaction with SUMO proteins. Here we revealed a marked reduction in SUMOylated proteins that localize to the XY body in *Setx*^*−/−*^ spermatocytes and that senataxin, which localizes preferentially to the XY body during the pachytene stage, is also SUMOylated. It is possible that SUMOylated senataxin has a role in DDR protein-mediated MSCI. Indeed, we showed that ATRIP localized predominantly to the axial elements of the XY chromosomes but when senataxin was disrupted there was a significant reduction of its association with the XY chromosomes. This might be explained by lack of SUMOylation. It is also evident from immunoblotting that in the absence of senataxin, the protein levels of ATRIP present was markedly reduced, providing an explanation for the reduced ATRIP SUMOylation observed in *Setx*^*−/−*^ spermatocytes. The lower levels of ATRIP and its markedly reduced recruitment to the axial elements of XY chromosomes are likely to contribute to the abnormality in ATR activity. Our data suggest that decreased levels of NEK1 and its mislocalization in *Setx*^*−/−*^ pachytene cells accounts for the reduced levels of ATRIP. Liu *et al.* [35] showed that even in undamaged cells, NEK1 is required for maintaining levels of ATRIP, the association between ATR and ATRIP, and the basal kinase activity of ATR [35]. In this way, NEK1 primes the ATR-ATRIP heterodimer for a robust DNA damage response. Thus, the defect in NEK1 and the consequent reduction in ATRIP protein levels observed in *Setx*^*−/−*^ pachytene spermatocytes could account for the defective activation and signaling through ATR leading to the failure of MSCI.

The great majority of SUMOylation staining was localized to the XY body and in the absence of senataxin, this is markedly reduced. As discussed above, ATRIP was one of several SUMOylated proteins associated with the XY body during meiosis. Others include ATRX, which is a chromatin remodeling protein shown recently to promote the incorporation of histone variant H3.3 at particular transcribed genes to facilitate transcriptional elongation through G-rich sequences [53]. This is of interest as we showed that senataxin is also SUMOylated and localizes to the XY body. The capacity of senataxin to resolve R-loops [13, 14, 19, 20] and the propensity of GC-rich repeats to form not only G-quadruplexes but also R-loops, leading to abortive transcription [54], supports a common goal for these two proteins in XY inactivation, ensuring that transcription is completed on the XY chromosomes before entry into the diplotene stage. Other SUMOylated proteins include DHX15, a DEAH-box helicase that is part of the splicesosome complex and contributes to the activation of signaling pathways [55]; RUVB-like ATPase that functions in chromatin decondensation [56] and YBOX transcription factors. Clearly, all of these proteins have the potential to be involved in remodeling chromatin on the XY chromosomes to ensure the onset of gene silencing or they could contribute to the re-emergence of transcriptional activation at later stage of meiosis.

The marked deficiency in the recruitment of CHD4 to the XY body in *Setx*^*−/−*^ pachytene cells and its role in transcriptional regulation substantiates further a central role for senataxin in gene silencing. CHD4 is a core component of the NuRD corepressor complex that remodels chromatin in a conformation refractory to active transcription [57, 58]. We provided evidence for the first time that CHD4 interacts with senataxin *in situ* on the XY body, which confirms the interaction between the two proteins demonstrated in proliferating cells using affinity chromatography, mass spectrometry and immunoblotting [16]. CHD4 has also been shown to interact with ATR [39]. In that study, ATR was shown to be in a complex that included CHD4, HDAC2 and other members of the NuRD complex, suggesting that as part of the complex ATR would have greater access to damaged DNA. CHD4 was subsequently found to localize to sites of DNA damage and cells depleted of this protein were hypersensitive to DNA damaging agents, exhibited cell cycle abnormalities and retained DNA DSBs for extended periods of time [59]. It was suggested that in CHD4-deficient cells, either the access of DNA repair proteins to the site of damage is compromised or that the deficiency of CHD4 leads to global alterations in higher-order chromatin structure, rendering it more vulnerable to accumulating DNA damage. Our data indicate that CHD4 plays a similar role in remodeling chromatin to allow access of silencing factors to asynapsed regions of the XY chromosomes to turn off or prevent transcription during meiosis. The defective recruitment of CHD4 to the XY body in *Setx*^*−/−*^ pachytene cells is expected to result in more open chromatin since it normally functions as a repressor of transcription [37, 46]. CHD4, a part of the NuRD histone deacetylase complex, is also involved in removing acetylation marks from histones to convert open transcriptionally active euchromatin into closed transcriptionally inactive heterochromatin. Indeed, higher levels of acetylated histones such as H3K9Ac and H4K16Ac were observed on the XY body in *Setx*^*−/−*^. Although H3K9Ac is present at low levels in the early stages of prophase 1, it increases strongly by midpachytene on autosomes but is excluded from XY chromosomes which are initiating MSCI [42]. H3K9Ac strongly decorated the XY body in *Setx*^*−/−*^ cells, which is consistent with a failure of transcription repression in *Setx*^*−/−*^ pachytene cells. Furthermore, H3K4me1, a marker of transcriptionally inactive heterochromatin, was excluded from the XY body in *Setx*^*−/−*^ spermatocytes, demonstrating the important role of senataxin in coordinating CHD4 recruitment, histone modification and transcriptional silencing. This is in agreement with the MSCI failure and abnormal expression of X- and Y-linked genes observed previously [20]. Although H3K4me1 has generally been associated with transcriptional activation, several reports have been published discussing its role in silencing. Page *et al.* [42] showed H3K4me1 localization to the XY body during the pachytene stage, which prompted the authors to suggest its involvement in transcriptional silencing during MSCI. Additionally, Cheng *et al.* [60] described its association with transcriptional silencing in embryonic fibroblasts, macrophages and human embryonic stem cells. Thus, there exists evidence suggesting both a role in transcriptional activation and silencing for H3K4me1, which may depend on the cell type investigated.

**Figure 8:**
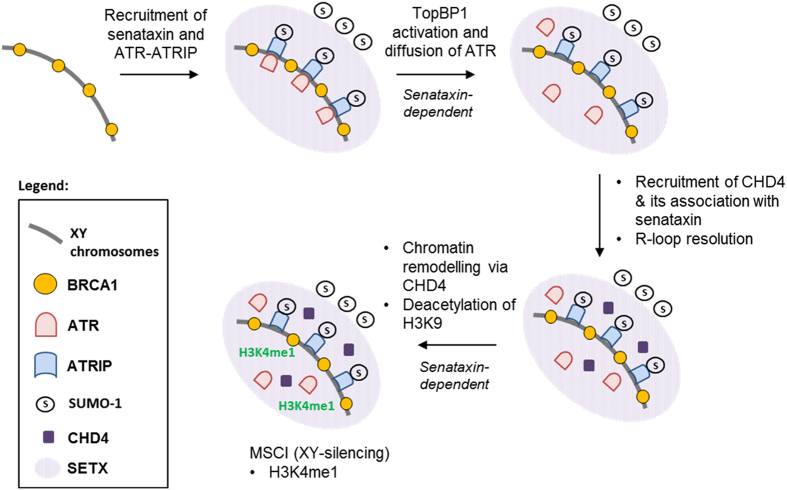
Integrated role of senataxin in meiotic sex chromosome inactivation (MSCI). MSCI is initiated with breast cancer type 1 susceptibility protein (BRCA1) first coating the axial element of the XY chromosomes during the pachytene stage. This is followed by recruitment of senataxin and ataxia telangiectasia and Rad3-related protein (ATR)-interacting partner (ATR/ATRIP) where both senataxin and ATRIP are present as SUMOylated proteins. Binding of TopBP1 activates ATR, which then disperses into the XY chromatin domain where it functions to phosphorylate other proteins, which may include histone H2AX (γH2AX). This step is senataxin-dependent. ATR diffusion into surrounding chromatin is not essential for H2AX phosphorylation as this occurs in the absence of senataxin where ATR is confined to the axial elements of XY chromosomes. Senataxin acts to resolve R-loops on XY chromosomes and associates with CHD4, which is part of the nucleosome remodeling complex (NuRD), to assist in the deacetylation of histones to downregulate active gene expression. The remodeling of chromatin for MSCI is dependent on senataxin. In addition, monomethylation of H3K4 occurs, all of which gives rise to heterochromatin formation and XY silencing.

We have provided important mechanistic insight into gene silencing of the XY chromosomes in meiosis and the central role of senataxin in this process, involving activation of ATR, recruitment of the chromatin remodeler CHD4 and the ensuing posttranslational histone modifications necessary for MSCI. These new data provided additional insight into the sequence of events involved in MSCI (Figure 8) at the pachytene stage. The initial step is BRCA1-dependent recruitment of ATR and that of senataxin as we have recently demonstrated [20]. In the absence of senataxin, ATR, ATRIP and TopBP1 are all retained on the axial element of the XY chromosomes. Additionally, the protein levels of both ATRIP and TopBP1 are reduced as well as their binding to the XY chromosomes. Under these conditions, we did not detect diffusion of ATR or its downstream substrates, although normal levels of H2AX phosphorylation were detected over the XY body. This suggests that another PIKK distinct from ATR can carry out this phosphorylation. Although γH2AX is present, it does not ensure that MSCI will occur, suggesting that it is not a single determinant but is instead only one of several required chromatin modifications to achieve gene silencing. We provided further insight into this by demonstrating the requirement for senataxin to recruit CHD4 to the XY chromosomes and that these two proteins interact at the level of the XY body. Specific evidence of a concerted action between senataxin and CHD4 on the XY chromosomes was demonstrated at the X-linked *Timp1* gene (Figure 7d). The higher levels of histone acetylation modifications, which were observed in *Setx*^*−/−*^ spermatocytes, have been previously associated with aberrant active gene transcription for X- and Y-linked genes during pachytene [20]. This is also in agreement with increased levels of R-loop formation observed in *Setx*^*−/−*^ spermatocytes, which may also contribute to the failure of MSCI and inhibition of meiosis [19, 20]. In summary, senataxin has been demonstrated to facilitate transcription termination by resolving R-loops [13], coordinate transcription with meiotic recombination [20], act at the interface of transcription, DNA damage response and RNA surveillance [5, 16] and protect against DNA DSBs generated in transcription-coupled DNA repair [61]. The findings in this report adds a new level of complexity to senataxin’s function in meiosis by demonstrating a direct role in modulating chromatin remodeling and epigenetic gene silencing via CHD4 and the ATR signaling pathway, and highlights its pivotal role in coordinating gene regulation and the DNA damage response.

## Materials and Methods

### Animal husbandry and genotyping

All animal experiments were approved by the QIMR Berghofer Medical Research Institute Animal Ethics Committee, The University of Queensland. The mice were weaned at 21 days postpartum and ear clipped for identification. Genotyping was carried out via polymerase chain reaction (PCR) on genomic DNA isolated from tail tips as described in Becherel *et al.* [20].

### Spermatocyte spreads

All spreads were made from testes collected from adult 35-day-old mice. Briefly, testes were decapsulated, finely chopped and rinsed in 1 ml of GIBCO serum-free DME medium (Life Technologies, Carlsbad, CA, USA) containing 1× Complete Protease Inhibitor (Roche, Basel, Switzerland). Six microliters of GIBCO serum-free DME medium (Life Technologies) was then added and tubes were allowed to stand on ice for 5 min to allow large clumps and cellular debris to settle. Six microliters of the remaining supernatant from each sample was subsequently aliquoted into Eppendorf tubes, with each tube containing 1 ml of suspension. Tubes were then centrifuged for 5 min at 5 000 *g* to pellet the cell suspension. The pellet was then resuspended in 0.1 m sucrose and applied onto glass slides prewetted with 1% paraformaldehyde and 0.1% Triton X-100 in phosphate-buffered saline (PBS). Cells were fixed on the glass slides and air-dried for 3 h at room temperature. The slides were subsequently washed with 1:250 Kodak Photo-Flo 200 (Kodak Professional, Rochester, NY, USA) in 1× PBS and air-dried for 1 h. Once dried, spreads were stored at −80 °C.

### Immunofluorescence

Spermatocyte spreads were blocked in 20% fetal calf serum, 2% bovine serum albumin (BSA), 0.2% Triton X-100 in 1× PBS for 1 h at room temperature. Slides were incubated overnight at 4 °C in a humidified chamber with the following primary antibodies diluted in blocking buffer: mouse monoclonal anti-SCP3 (Abcam, Cambridge, MA, USA; AB97672) at a 1:100 dilution, rabbit anti-SCP3 (Novus Biologicals, Littleton, CO, USA; NB300-230) at a 1:100 dilution, goat anti-ATR (Santa Cruz Biotechnology, Dallas, TX, USA; SC-1887) at a 1:100 dilution, rabbit anti-NEK1 (Bioss Inc., Woburn, MA, USA; BS-7814R) at a 1:100 dilution, rabbit anti-ATRIP (Thermo Scientific, Waltham, MA, USA; PA1-519) at a 1:100 dilution, mouse monoclonal anti-CHK1 (Cell Signaling Technology, Danvers, MA, USA; 2 360) at a 1:100 dilution, rabbit anti-CHD4 (Genetex, Irvine, CA, USA; GTX124186), sheep anti-Setx (in-house) at a 1:300 dilution, rabbit anti-TopBP1 (Abcam; AB2402) at a 1:100 dilution, rabbit anti SUMO-1 (Abcam; AB32058) at a 1:100 dilution, rabbit anti-SUMO-2/3 (Abcam; AB3742) at a 1:100 dilution, rabbit anti-phospho ATR (S428) (Cell Signaling Technology; 2 853) at a 1:100 dilution, rabbit anti-phospho RPA (S33) (Bethyl Laboratories Inc., Montgomery, TX, USA; A300-246A) at a 1:100 dilution, rabbit anti-phospho CHK1 (S317) (Cell Signaling Technology; 2 344) at a 1:100 dilution, rabbit anti-H3K4me1 (Abcam; AB8895) at a 1:100 dilution, rabbit anti-H3K9Ac (Bioss Inc.; BS-3776A) at a 1:100 dilution, and rabbit anti-H4K12Ac (Bioss Inc.; BS-3746R) at a 1:100 dilution. Slides were then rinsed three times with 1× PBS containing 0.5% Triton X-100 for 5 min each at room temperature on an orbital shaker. Slides were then incubated for 1 h at 37 °C in a humidified chamber with the following Alexa-Dye-conjugated secondary antibodies (Life Technologies) diluted 1:250 in blocking buffer: Alexa 488 chicken anti-mouse (A-21200), Alexa 594 donkey anti-rabbit (A-11032), Alexa 594 donkey anti-goat (A-11058) and Alexa 350 goat anti-mouse (A-11045). Subsequently, slides were washed three times as before and Hoechst 33342 (1:10 000) (Life Technologies) was applied for 10 min to stain nuclei. Slides were finally washed two times and glass coverslips were mounted for imaging. Images were captured using a digital camera (AxioCam Mrm; Carl Zeiss Microimaging Inc., Jena, Germany) attached to a fluorescent microscope (Axioskop 2 mot plus; Carl Zeiss Microimaging) equipped with the appropriate filters, and the AxioVision 4.8 software (Carl Zeiss Microimaging Inc.). The objective used was an EC Plan-Neofluar 10x/0.3 (Carl Zeiss Microimaging Inc.). Images were subsequently assembled in Adobe Photoshop 5 (Adobe Systems, San Jose, CA, USA), and contrast and brightness were adjusted on the whole image panel at the same time. Fluorescence intensity was quantitated on at least 50 individual cells per marker and condition using the RAW images and NIH ImageJ software version 1.47 (NIH, Bethesda, MD, USA). Statistical analysis was carried out using Student’s *t*-test.

### Proximity ligation assay

PLA (Duolink; Olink Bioscience, Uppsala, Sweden) was performed according to the manufacturer's protocol with the following primary antibodies: mouse monoclonal anti-SCP3 (Abcam; AB97672) at a 1:100 dilution, rabbit anti SUMO-1 (Abcam; AB32058) at a 1:100 dilution, rabbit anti-phospho ATR (S428) (Cell Signaling Technology; 2 853) at a 1:100 dilution, sheep anti-Setx (in-house) at a 1:300 dilution, rabbit anti-CHD4 (Abcam; AB72418), and rabbit anti-BRCA1 (with courtesy of Prof. David Livingstone, Dana-Farber Cancer Institute, Harvard Medical School, Boston, MA, USA) at a 1:300 dilution. Corresponding PLA PLUS and MINUS probes were subsequently applied. Slide mounting and imaging was performed as described above.

### Immunoprecipitation of SUMOylated proteins from *Setx*^*+/+*^ and *Setx*^*−/−*^ testes

Testes of 35-day-old mice (ground with a pestle to disrupt their structure) were lysed for 1 h at 4 °C on a rotating wheel with 1 ml of lysis buffer (50 mM Tris-HCl, pH 7.4, 150 mM NaCl, 2 mM EGTA, 2 mM EDTA, 25 mM β-glycerophosphate, 0.1 mM Na_3_VO_4_, 0.1 mM PMSF, 25 mM NaF, 0.2% Triton X-100, 0.3% NP-40 and 1× EDTA-free Complete Protease Inhibitor (Roche) 25 μM NEM (Sigma-Aldrich, St Louis, MO, USA)). Cellular debris was pelleted by centrifugation at 16 100 *g* at 4 °C for 10 min, and protein concentration was determined using Lowry Assay (Bio-Rad Laboratories Inc., Hercules, CA, USA). Three micrograms of total cell extract were precleared with 20 μl of protein A beads (50:50 slurry) (Merck Millipore, Billerica, MA, USA) for 3 h at 4 °C on a rotating wheel. Extracts were centrifuged for 5 min at 2 000 *g*, beads were removed and 10 μg of anti-SUMO antibody (Abcam) was added to the extract. Extracts and antibody were incubated overnight at 4 °C on a rotating wheel to allow binding of the antibody to SUMOylated protein. The next day, 40 μl of protein A beads (50:50 slurry) (Merck Millipore) was added to the extract and incubated for 2 h at 4 °C on a rotating wheel. Extracts were centrifuged for 5 min at 2 000 *g* and the beads were removed. The immunoprecipitate was subsequently washed three times with lysis buffer and the beads were resuspended in SDS loading buffer containing 2× DTT and separated via 4–12% SDS-PAGE using Novex Tris-Glycine gels (Life Technologies) at 110 V for 1.5 h. Once separated, gel was stained with G-250 Colloidal Coomassie (Life Technologies) overnight. Gel was subsequently destained using Milli-Q water.

### Tryptic digestion of SUMOylated proteins from mice spermatocytes for LC-MS/MS

Bands were excised from gel using a sterile scalpel and were left to destain (50% acetonitrile, 50% ammonium bicarbonate) for 4 h. Trypsin (Promega, Madison, WI, USA) was added 1:20 by weight in 50 mM ammonium bicarbonate until gel pieces were covered and left to digest overnight. Subsequently, 100% acetonitrile was added and tube was vortexed for 10 min. Liquid in the tube was then transferred into a fresh Eppendorf tube. Fifty percent acetonitrile was added to the gel pieces and vortexed again for 10 min. Liquid was combined with the liquid collected in the previous step. Tubes were spun in a Speedy-Vac for 30 min to dry liquid to ~5 μl. This volume was reconstituted to 20 μl 0.1% trifluoroacetic acid (TFA) and applied to a C18 STAGE tip (Thermo Fisher Scientific, Waltham, MA, USA) by pipetting up and down for ~10 times. Tips were then washed with 10 μl of 0.1% TFA and peptides were eluted with 50% acetonitrile intro a fresh Eppendorf tube. Eluant was frozen at −20 °C and shipped to Dr Mark E Graham (Children’s Medical Research Institute, University of Sydney, NSW, Australia) for liquid chromatography mass spectrometry analyses.

### Liquid chromatography mass spectrometry identification of proteins immunoprecipitated from mice spermatocytes

Coomassie-stained protein bands from the anti-SUMO-1 immunoprecipitation SDS-PAGE gel were excised using a sterile scalpel and were destained (50% acetonitrile, 50% ammonium bicarbonate) for 4 h. Trypsin (Promega) was added 1:20 protein:trypsin by weight in 50 mM ammonium bicarbonate until gel pieces were covered and digested for 16 h. To extract the tryptic peptides, the volume was increased by 50% with acetonitrile, the tube was vortexed for 10 min and the liquid was then transferred into a fresh tube. An additional solution of 50% acetonitrile in water was added to the gel pieces, the tube was vortexed for 10 min and the liquid was combined with the liquid from the previous step. Tubes were spun in a Speedy-Vac for 30 min to dry liquid to ~5 μl. This volume was diluted to 20 μl 0.1% TFA and applied to a C18 STAGE tip (Thermo Fisher Scientific) by pipetting up and down for ~10 times. Tips were then washed with 10 μl of 0.1% TFA and peptides were eluted with 50% acetonitrile into a tube for storage at −20 °C. One-half of each sample was analyzed by LC-MS/MS. A Dionex 3 000 Ultimate HPLC system was used (Dionex/Thermo Fisher Scientific, Scoresby, VIC, Australia). Samples were loaded onto a trap column (300 μm inside diameter, 1 mm long; Acclaim PepMap100 C18, 5 μm, Thermo Fisher Scientific) for 5 min at 5 μl min^−1^ with phase A (0.1% formic acid in water). The sample was eluted through a 16 cm long, 75 μm inside diameter analytical column (3 μm 120 Å Reprosil Pur C18-AQ; Dr Maisch Gmbh, Ammerbuch-Entringen, Germany) at 250 nl min^−1^. The gradient was from 0 to 35% phase B (90% acetonitrile, 9.9% water and 0.1% formic acid) in 65 min. Phase B was increased to 100% in 5 min and held at 100% for 5 min. The column eluate was sprayed into an LTQ Velos orbitrap mass spectrometer (Thermo Fisher Scientific) through a 10 μm inside diameter SilicaTip (New Objective) with 2.2 kV applied. Up to seven of the most intense peptide precursor ions above a threshold of 5 000 counts were selected in the range *m*/*z* 300–1 500 for MS/MS for 90 min after the start of the gradient. Both MS and MS/MS detection was in the orbitrap at a resolution of 30 000 and 7 500, respectively. Detailed parameters were as follows: isolation width was 2 (*m*/*z*), normalized collision energy was 40, activation time was 0.1 ms, +1 charge state ions were rejected, ions within 100 ppm of previously selected ions were excluded from MS/MS for 10 s, MS target was 1 000 000 counts for up to 50 ms and MS/MS target was 50 000 for up to 300 ms. All MS raw data was submitted via Proteome Discoverer 1.3 (Thermo Fisher Scientific) for database searching with Mascot 2.4 (Matrix Science, London, UK). The database was UniProtKB (downloaded July 2013, *Mus musculus*, 16 673 entries). Precursor ion tolerance was 10 p.p.m. and fragment ion tolerance was 0.05 Da. Variable modifications were deamidation (NQ) and oxidation (M) and maximum missed trypsin cleavages was 3 (trypsin cleavage before proline was allowed). The Mascot result files were imported to Scaffold 4.4.1 (Proteome Software, OR, USA). Only proteins with at least two unique peptides were considered. Protein false discovery rate was 0.1% and peptide false discovery rate was 0.02%. Minimum protein probability of identification was 99% (*P*<0.01) and minimum peptide probability of identification was 95% (*P*<0.05).

### Immunoblotting for DDR proteins from *Setx*^*+/+*^ and *Setx*^*−/−*^ testes

Testes of 35-day-old mice (ground with a pestle to disrupt their structure) were lysed for 1 h at 4 °C on a rotating wheel with 1 ml of lysis buffer (50 mM Tris-HCl, pH 7.4, 150 mM NaCl, 2 mM EGTA, 2 mM EDTA, 25 mm β-glycerophosphate, 0.1 mM Na_3_VO_4_, 0.1 mm PMSF, 25 mm NaF, 0.2% Triton X-100, 0.3% NP-40 and 1× EDTA-free Complete Protease Inhibitor (Roche)). Cellular debris was pelleted by centrifugation at 16 100 *g* at 4 °C for 10 min, and protein concentration was determined using Lowry Assay (Bio-Rad). Thirty micrograms of total cell extract were resuspended in SDS loading buffer containing 2× DTT and separated via 4–12% SDS-PAGE using Novex Tris-Glycine gels (Life Technologies) at 110 V for 1.5 h. The proteins were then transferred onto a Hybond-C nitrocellulose membrane (Pall Life Sciences, NY, USA) using a high-molecular-weight transfer buffer (100 mM Tris, 40 mM glycine, 0.05% SDS, 20% methanol) at 100 V for 1 h at 4 °C in a mini-protean gel rig (Bio-Rad). Membranes were the blocked in 5% skim milk in TBS/Tween-20 or 5% BSA/TBS/Tween-20 for 1 h and immunoblotting was performed with the following primary antibodies diluted 1:1 000 in 5% skim milk/TBS/Tween-20 or 5% BSA/TBS/Tween-20 at 4 °C overnight: goat anti-ATR (Santa Cruz Biotechnology; SC-1887), rabbit anti-NEK1 (Bioss Inc., BS-7814R), rabbit anti-ATRIP (Thermo Scientific; PA1-519), mouse monoclonal anti-CHK1 (Cell Signaling Technology; 2 360), rabbit anti-CHD4 (Abcam; AB72418), sheep anti-Setx (in-house), rabbit anti-TopBP1 (Abcam; AB2402), rabbit anti SUMO-1 (Abcam; AB32058), rabbit anti-phospho ATR (S428) (Cell Signaling Technology; 2 853), rabbit anti-glyceraldehyde 3-phosphate dehydrogenase (Genetex; GTX100118) and rabbit anti-α tubulin (Genetex; GTX 112141). Membranes were subsequently washed thee times with TBS/Tween-20 for 5 min each and incubated with the following secondary antibodies conjugated with horseradish peroxidase (HRP) diluted 1:5 000 in 5% skim milk/TBS/Tween-20 or 5% BSA/TBS/Tween- 20 for 1 h at room temperature: HRP-conjugated donkey anti-sheep (Merck Millipore; AP184P), HRP-conjugated donkey anti-mouse (Merck Millipore; AP192P) and HRP-conjugated donkey anti-rabbit (Merck Millipore; AP182P). Membranes were then analyzed using the Western Lightning Plus Enhanced Chemiluminescence (ECL) reagent (Perkin-Elmer, Waltham, MA, USA).

### Reverse transcription-PCR and gene expression analysis

Total RNA was isolated from 35-day-old mice testes using the RNeasy Mini Kit (Qiagen, Valencia, CA, USA) according to the manufacturer's protocol. cDNA synthesis was performed as described previously [20]. Primer pairs used for reverse transcription-PCR gene expression analyses are detailed in Supplementary Table S2.

### ChIP assay

ChIP assays were performed as described previously [12]. Briefly, crosslinking mix (11% formaldehyde, 100 mM NaCl, 0.5 mM EGTA, 50 mM HEPES, pH 8.0, supplemented with protease and phosphatase inhibitors) was added to testes of 35-day-old mice (ground with a pestle to disrupt their structure). Crosslinking was quenched after 15 min with 1.25 M glycine and lysed with ChIP lysis buffer (1% SDS, 10 mm EDTA, pH 8.0, 50 mM Tris-HCl, pH 8.0, supplemented with protease and phosphatase inhibitors) for 30 min at 4 °C. DNA was sheared by sonication and insoluble components were removed by centrifugation. Two milligrams of protein was diluted to 600 ml with dilution buffer (1% Triton X-100, 150 mM NaCl, 2 mM EDTA, pH 8.0, 20 mM Tris-HCl, pH 8.0, supplemented with protease and phosphatase inhibitors) and precleared with protein A-Sepharose/G-Sepharose beads (Amersham Biosciences, Buckinghamshire, UK) and 4 μg of salmon sperm DNA. Senataxin and CHD4 was immunoprecipitated separately using 2 μg of antibody overnight at 4 °C. DNA fragments were then eluted in elution buffer (1% SDS, 100 mM NaHCO_3_), purified and analyzed by PCR. Primer pairs used for *Timp1* are detailed in Supplementary Table S2. PCR products were subsequently quantitated using ImageJ 1.48v (NIH, Bethesda, MD, USA). Results for each experiment were calculated relative to the input. Error bars show the standard deviation and significance was determined using the Student's *t*-test.

### RLFS prediction and DRIP assay

An RLFS was predicted (Dr Thidathip Wongsurawat, personal communication) in the 5′-UTR region of *Timp1*. This region was then used for DRIP analysis. Genomic DNA and RNA extraction from mice testes were performed using the DNeasy Blood and Tissue Kit (Qiagen) following the manufacturer's instructions. DRIP assay was then performed as described previously [19]. Primer pairs for *Timp1* are detailed in Supplementary Table S2. PCR products were subsequently quantitated using ImageJ 1.48v. Results for each experiment were calculated relative to the input.
