# Supplementary material for: Senataxin controls meiotic silencing through ATR activation and chromatin remodeling
Source: Cell Discov. 2015 Sep 29;1:15025–. doi: 10.1038/celldisc.2015.25 (PMC4860845; doi:10.1038/celldisc.2015.25)
Supplement: Supplementary Figure S1 [file celldisc201525-s1.pdf]

Supplementary Figure 1

A

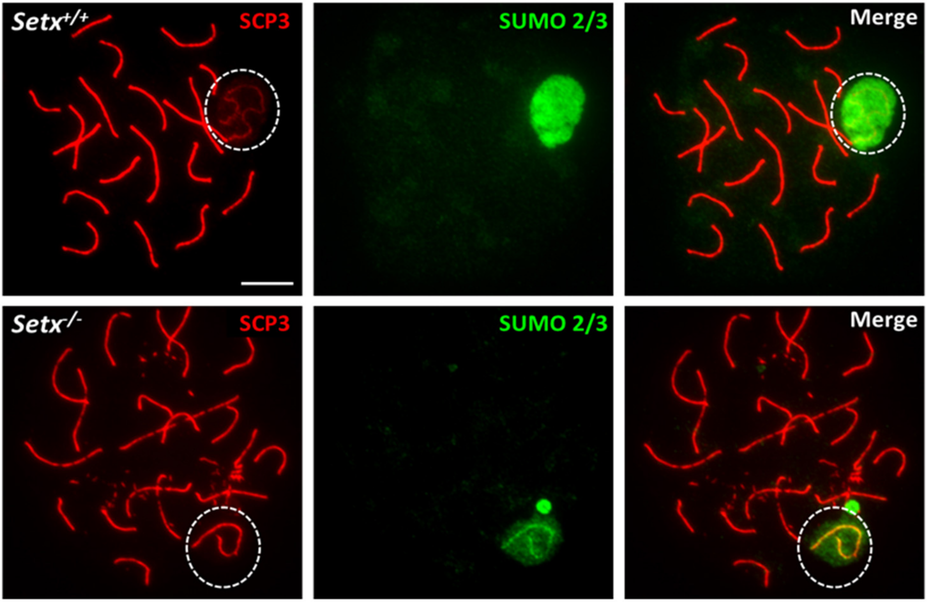

B

ID	Position	Peptide	Score	Cutoff	Type
gi|160184873|sp|A2AKX3.1|SETX_MOUSE RecName: Full=Probable helicase senataxin; AltName: Full=Amyotrophic lateral sclerosis 4 protein homolog; AltName: Full=SEN1 homolog	78	SHFEKSMKAEAEDDD	30.537	19.705	Sumoylation
gi|160184873|sp|A2AKX3.1|SETX_MOUSE RecName: Full=Probable helicase senataxin; AltName: Full=Amyotrophic lateral sclerosis 4 protein homolog; AltName: Full=SEN1 homolog	863	SLFKKEIKSEELDNS	45.799	19.705	Sumoylation
gi|160184873|sp|A2AKX3.1|SETX_MOUSE RecName: Full=Probable helicase senataxin; AltName: Full=Amyotrophic lateral sclerosis 4 protein homolog; AltName: Full=SEN1 homolog	998 - 1002	AATCRGQVIVISDSDEEED	85.234	63.668	SUMO Interaction
gi|160184873|sp|A2AKX3.1|SETX_MOUSE RecName: Full=Probable helicase senataxin; AltName: Full=Amyotrophic lateral sclerosis 4 protein homolog; AltName: Full=SEN1 homolog	1051	SVEKQLVKEEERYPV	45.307	19.705	Sumoylation
gi|160184873|sp|A2AKX3.1|SETX_MOUSE RecName: Full=Probable helicase senataxin; AltName: Full=Amyotrophic lateral sclerosis 4 protein homolog; AltName: Full=SEN1 homolog	1438 - 1442	SGGVPTDVVMVSASEDPVD	64.429	63.668	SUMO Interaction
gi|160184873|sp|A2AKX3.1|SETX_MOUSE RecName: Full=Probable helicase senataxin; AltName: Full=Amyotrophic lateral sclerosis 4 protein homolog; AltName: Full=SEN1 homolog	2154 - 2158	GGVPFSCVIVDEAGQSCEV	73.943	63.668	SUMO Interaction
