# Supplementary figures and images for: Senataxin controls meiotic silencing through ATR activation and chromatin remodeling
Source: Cell Discov. 2015 Sep 29;1:15025–. doi: 10.1038/celldisc.2015.25 (PMC4860845; doi:10.1038/celldisc.2015.25)

## Supplementary Figure 2

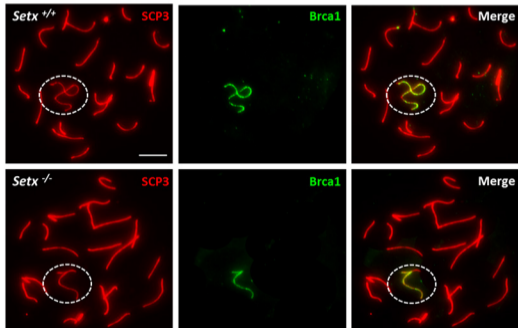

Supplement: Supplementary Figure S2 [file celldisc201525-s2.pdf]

## Supplementary Figure 3

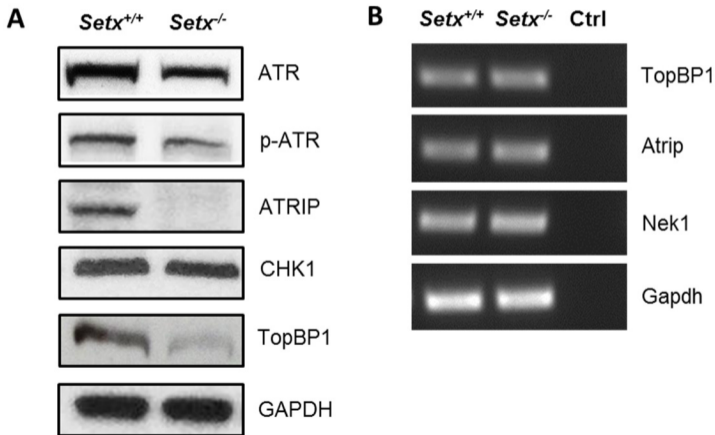

Supplement: Supplementary Figure S3 [file celldisc201525-s3.pdf]

**Supplementary Figure 4**

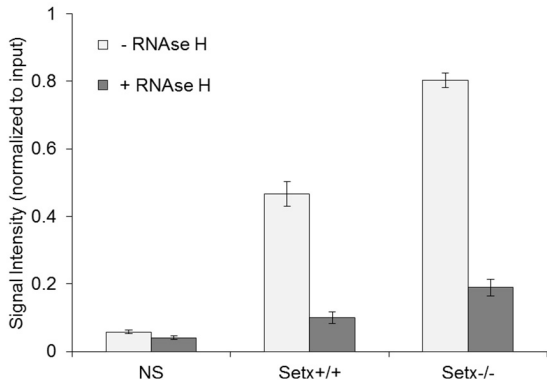

Supplement: Supplementary Figure S4 [file celldisc201525-s4.pdf]

## Supplementary Figure 5

A

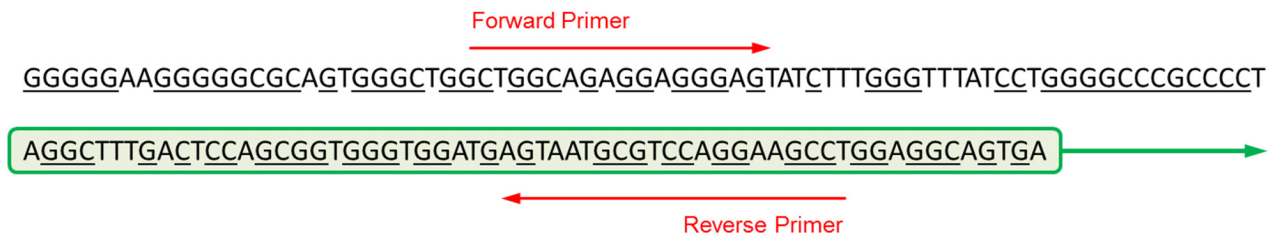

B

**Timp1**

tissue inhibitor of metalloproteinase 1

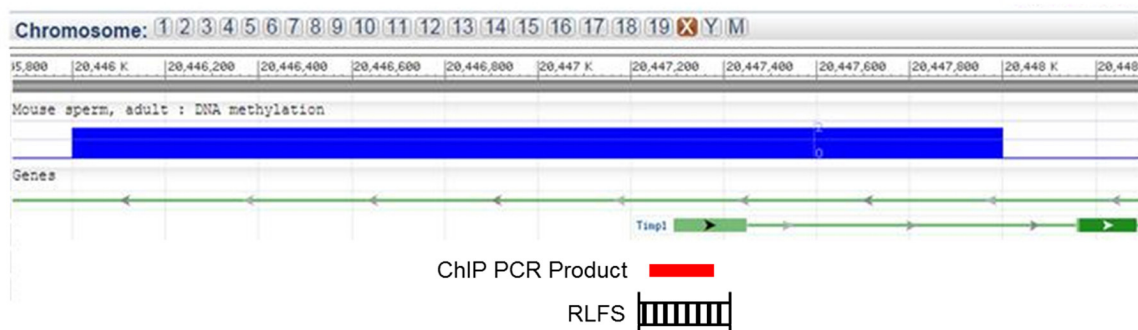

Supplement: Supplementary Figure S5 [file celldisc201525-s5.pdf]
