# Supplementary material for: Senataxin controls meiotic silencing through ATR activation and chromatin remodeling
Source: Cell Discov. 2015 Sep 29;1:15025–. doi: 10.1038/celldisc.2015.25 (PMC4860845; doi:10.1038/celldisc.2015.25)
Supplement: Supplementary Table S1 [file celldisc201525-s6.pdf]

Protein name	Protein accession numbers	Protein molecular weight (Da)	Protein identification probability	p	Number of unique peptides	Number of unique spectra	Number of total spectra	Percentage sequence coverage
X-ray repair cross-complementing protein 5 OS=Mus musculus GN=Xrcc5 PE=2 SV=4	XRCC5_MOUSE	83,059.00	100.00%	0.002	5	5	6	7.10%
Interleukin enhancer-binding factor 3 OS=Mus musculus GN=Ilf3 PE=1 SV=2	ILF3_MOUSE	96,021.90	100.00%	0.002	5	7	8	6.79%
Putative pre-mRNA-splicing factor ATP-dependent RNA helicase DHX15 OS=Mus musculus GN=Dhx15 PE=2 SV=2	DHX15_MOUSE	91,009.30	100.00%	0.002	35	54	80	41.60%
E3 UFM1-protein ligase 1 OS=Mus musculus GN=Ufl1 PE=1 SV=2	UFL1_MOUSE	89,522.40	100.00%	0.002	30	38	43	41.00%
ATP-dependent RNA helicase DDX1 OS=Mus musculus GN=Ddx1 PE=1 SV=1	DDX1_MOUSE	82,501.10	100.00%	0.002	28	38	42	42.00%
Splicing factor, proline- and glutamine-rich OS=Mus musculus GN=Sfpq PE=1 SV=1	SFPQ_MOUSE	75,441.90	100.00%	0.002	25	33	35	30.90%
Spermatogenesis-associated protein 20 OS=Mus musculus GN=Spata20 PE=2 SV=1	SPT20_MOUSE	88,473.10	100.00%	0.002	25	43	65	38.90%
RNA-binding protein MEX3D OS=Mus musculus GN=Mex3d PE=2 SV=2	MEX3D_MOUSE	65,329.20	100.00%	0.002	7	9	10	16.30%
E3 ubiquitin-protein ligase Trim36 OS=Mus musculus GN=Trim36 PE=1 SV=2	TRI36_MOUSE	82,792.60	100.00%	0.002	7	7	7	13.40%
Heterogeneous nuclear ribonucleoprotein U-like protein 1 OS=Mus musculus GN=Hnrnpul1 PE=1 SV=1	HNRL1_MOUSE	96,003.10	100.00%	0.002	5	7	7	5.94%
Eukaryotic translation initiation factor 3 subunit B OS=Mus musculus GN=Eif3b PE=1 SV=1	EIF3B_MOUSE	91,371.20	100.00%	0.002	5	5	5	8.84%
Small ubiquitin-related modifier 1 OS=Mus musculus GN=Sumo1 PE=1 SV=1	SUMO1_MOUSE	11,557.00	100.00%	0.002	4	5	6	33.70%
Ran GTPase-activating protein 1 OS=Mus musculus GN=Rangap1 PE=1 SV=2	RAGP1_MOUSE	63,532.70	100.00%	0.002	4	4	4	8.66%
ATR-interacting protein OS=Mus musculus GN=Atrip PE=1 SV=2	ATRIP_MOUSE	85,548.00	100.00%	0.002	3	3	3	3.82%
RAD50-interacting protein 1 OS=Mus musculus GN=Rint1 PE=2 SV=2	RINT1_MOUSE	90,096.80	100.00%	0.002	3	3	4	4.04%
Nucleolin OS=Mus musculus GN=Ncl PE=1 SV=2	NUCL_MOUSE	76,724.40	100.00%	0.002	3	3	3	7.64%
Gametogenetin OS=Mus musculus GN=Ggn PE=1 SV=2	GGN_MOUSE	68,891.90	100.00%	0.002	3	3	3	5.79%
WW domain-binding protein 11 OS=Mus musculus GN=Wbp11 PE=1 SV=2	WBP11_MOUSE	69,876.80	99.80%	0.002	2	2	2	2.81%
Elongation factor 1-alpha 1 OS=Mus musculus GN=Eef1a1 PE=1 SV=3	EF1A1_MOUSE	50,114.20	99.80%	0.002	2	2	2	7.36%
E3 SUMO-protein ligase RanBP2 OS=Mus musculus GN=Ranbp2 PE=1 SV=2	RBP2_MOUSE	3,41,121.20	100.00%	0.002	135	184	264	43.50%
E3 ubiquitin-protein ligase HUWE1 OS=Mus musculus GN=Huwe1 PE=1 SV=5	HUWE1_MOUSE	4,82,635.70	100.00%	0.002	30	37	42	9.85%
Transcriptional regulator ATRX OS=Mus musculus GN=Atrx PE=1 SV=3	ATRX_MOUSE	2,78,590.20	100.00%	0.002	23	27	29	9.17%
Mediator of DNA damage checkpoint protein 1 OS=Mus musculus GN=Mdc1 PE=1 SV=1	MDC1_MOUSE	1,84,667.00	100.00%	0.002	18	18	21	14.90%
Small ubiquitin-related modifier 1 OS=Mus musculus GN=Sumo1 PE=1 SV=1	SUMO1_MOUSE	11,557.00	100.00%	0.002	9	12	21	52.50%
Heterogeneous nuclear ribonucleoproteins A2/B1 OS=Mus musculus GN=Hnrnpa2b1 PE=1 SV=2	ROA2_MOUSE	37,403.30	100.00%	< 0.001	15	20	86	46.50%
Proliferating cell nuclear antigen OS=Mus musculus GN=Pcna PE=1 SV=2	PCNA_MOUSE	28,785.50	100.00%	< 0.001	10	15	41	42.10%
Small ubiquitin-related modifier 2 OS=Mus musculus GN=Sumo2 PE=2 SV=1	SUMO2_MOUSE	10,871.20	100.00%	< 0.001	4	4	9	35.80%
Apolipoprotein E OS=Mus musculus GN=Apoe PE=1 SV=2	APOE_MOUSE	35,866.30	100.00%	< 0.001	4	5	5	10.90%
Serine/arginine-rich splicing factor 7 OS=Mus musculus GN=Srsf7 PE=1 SV=1	SRSF7_MOUSE	30,818.70	100.00%	< 0.001	4	4	8	13.10%
E3 SUMO-protein ligase NSE2 OS=Mus musculus GN=Nsmce2 PE=2 SV=1	NSE2_MOUSE	28,231.60	100.00%	< 0.001	3	4	5	8.91%
Exosome complex component RRP4 OS=Mus musculus GN=Exosc2 PE=2 SV=1	EXOS2_MOUSE	32,632.10	100.00%	< 0.001	3	3	3	9.22%
Replication protein A 32 kDa subunit OS=Mus musculus GN=Rpa2 PE=1 SV=1	RFA2_MOUSE	29,718.20	100.00%	< 0.001	3	3	3	11.10%
Mediator of RNA polymerase II transcription subunit 4 OS=Mus musculus GN=Med4 PE=2 SV=1	MED4_MOUSE	29,782.10	100.00%	< 0.001	3	3	3	11.10%
Splicing factor U2AF 35 kDa subunit OS=Mus musculus GN=U2af1 PE=1 SV=4	U2AF1_MOUSE	27,815.20	100.00%	< 0.001	2	4	12	8.79%
Methylosome subunit pICln OS=Mus musculus GN=Clns1a PE=1 SV=1	ICLN_MOUSE	26,021.10	99.80%	< 0.001	2	2	7	8.05%
Thioredoxin-related transmembrane protein 2 OS=Mus musculus GN=Tmx2 PE=2 SV=1	TMX2_MOUSE	33,944.00	99.80%	< 0.001	2	2	2	6.44%
Transcription initiation factor TFIID subunit 9 OS=Mus musculus GN=Taf9 PE=2 SV=1	TAF9_MOUSE	28,980.00	99.80%	< 0.001	2	2	2	7.95%
E3 SUMO-protein ligase RanBP2 OS=Mus musculus GN=Ranbp2 PE=1 SV=2	RBP2_MOUSE	3,41,121.20	100.00%	< 0.001	102	124	152	35.20%
Mediator of DNA damage checkpoint protein 1 OS=Mus musculus GN=Mdc1 PE=1 SV=1	MDC1_MOUSE	1,84,667.00	100.00%	< 0.001	17	17	19	13.80%
Elongation factor 1-alpha 1 OS=Mus musculus GN=Eef1a1 PE=1 SV=3	EF1A1_MOUSE	50,114.20	100.00%	< 0.001	16	20	24	27.70%
E3 ubiquitin-protein ligase UBR4 OS=Mus musculus GN=Ubr4 PE=1 SV=1	UBR4_MOUSE	5,72,290.60	100.00%	< 0.001	13	14	14	3.19%
DNA damage-binding protein 1 OS=Mus musculus GN=Ddb1 PE=1 SV=2	DDB1_MOUSE	1,26,855.10	100.00%	< 0.001	4	4	4	5.88%
Pre-mRNA-processing-splicing factor 8 OS=Mus musculus GN=Prpf8 PE=1 SV=2	PRP8_MOUSE	2,73,624.50	100.00%	< 0.001	4	4	4	2.01%
Ubiquitin-associated domain-containing protein 2 OS=Mus musculus GN=Ubac2 PE=2 SV=1	UBAC2_MOUSE	39,058.00	100.00%	< 0.001	3	4	4	11.90%
Probable E3 ubiquitin-protein ligase MYCBP2 OS=Mus musculus GN=Mycbp2 PE=1 SV=2	MYCB2_MOUSE	5,17,734.10	99.80%	< 0.001	2	2	2	0.43%
E3 ubiquitin-protein ligase synoviolin OS=Mus musculus GN=Syvn1 PE=1 SV=3	SYVN1_MOUSE	67,297.70	99.80%	< 0.001	2	2	2	2.29%
E3 ubiquitin-protein ligase HERC2 OS=Mus musculus GN=Herc2 PE=1 SV=3	HERC2_MOUSE	5,27,452.60	99.80%	< 0.001	2	2	2	0.54%
Huntingtin OS=Mus musculus GN=Htt PE=1 SV=2	HD_MOUSE	3,44,689.10	99.80%	< 0.001	2	2	2	0.77%
Heterogeneous nuclear ribonucleoproteins A2/B1 OS=Mus musculus GN=Hnrnpa2b1 PE=1 SV=2	ROA2_MOUSE	37,403.30	100.00%	< 0.001	31	52	92	61.50%
14-3-3 protein zeta/delta OS=Mus musculus GN=Ywhaz PE=1 SV=1	1433Z_MOUSE	27,771.90	100.00%	< 0.001	15	25	31	50.60%
THO complex subunit 4 OS=Mus musculus GN=Alyref PE=1 SV=3	THOC4_MOUSE	26,940.80	100.00%	< 0.001	14	25	38	59.20%
Synaptonemal complex protein 3 OS=Mus musculus GN=Sycp3 PE=1 SV=1	SYCP3_MOUSE	29,347.20	100.00%	< 0.001	9	12	15	33.50%
Eukaryotic translation initiation factor 4H OS=Mus musculus GN=Eif4h PE=1 SV=3	IF4H_MOUSE	27,341.90	100.00%	< 0.001	9	11	11	33.10%
GTP-binding nuclear protein Ran OS=Mus musculus GN=Ran PE=1 SV=3	RAN_MOUSE	24,423.10	100.00%	< 0.001	9	10	10	42.10%
Ubiquitin-conjugating enzyme E2 R1 OS=Mus musculus GN=Cdc34 PE=1 SV=1	UB2R1_MOUSE	26,622.20	100.00%	< 0.001	8	9	11	25.10%
Histone H1t OS=Mus musculus GN=Hist1h1t PE=1 SV=4	H1T_MOUSE	21,541.50	100.00%	< 0.001	8	11	13	36.50%
Heterogeneous nuclear ribonucleoprotein A3 OS=Mus musculus GN=Hnrnpa3 PE=1 SV=1	ROA3_MOUSE	39,652.20	100.00%	< 0.001	7	9	10	24.30%
Ubiquitin-conjugating enzyme E2 R2 OS=Mus musculus GN=Ube2r2 PE=2 SV=1	UB2R2_MOUSE	27,166.80	100.00%	< 0.001	4	5	5	23.10%
Geminin OS=Mus musculus GN=Gmnn PE=1 SV=1	GEMI_MOUSE	23,299.70	100.00%	< 0.001	3	4	4	17.00%
mRNA turnover protein 4 homolog OS=Mus musculus GN=Mrto4 PE=2 SV=1	MRT4_MOUSE	27,546.60	100.00%	< 0.001	3	4	4	14.20%
Spliceosome-associated protein CWC15 homolog OS=Mus musculus GN=Cwc15 PE=1 SV=1	CWC15_MOUSE	26,625.30	100.00%	< 0.001	3	3	3	16.60%
Mediator of RNA polymerase II transcription subunit 8 OS=Mus musculus GN=Med8 PE=2 SV=1	MED8_MOUSE	29,199.40	100.00%	< 0.001	3	3	3	17.90%
Chromatin target of PRMT1 protein OS=Mus musculus GN=Chtop PE=1 SV=2	CHTOP_MOUSE	26,586.10	100.00%	< 0.001	2	3	3	10.40%
Survival of motor neuron-related-splicing factor 30 OS=Mus musculus GN=Smndc1 PE=2 SV=1	SPF30_MOUSE	26,753.40	100.00%	< 0.001	2	3	4	8.40%
Elongation factor 1-beta OS=Mus musculus GN=Eef1b PE=1 SV=5	EF1B_MOUSE	24,694.10	99.80%	< 0.001	2	2	2	8.89%
X-linked lymphocyte-regulated protein PM1 OS=Mus musculus GN=Xlr PE=2 SV=1	XLR_MOUSE	24,613.00	99.80%	< 0.001	2	2	2	11.10%
Exosome complex component MTR3 OS=Mus musculus GN=Exosc6 PE=1 SV=1	EXOS6_MOUSE	28,370.60	99.80%	< 0.001	2	2	2	8.42%
Matrix-remodeling-associated protein 7 OS=Mus musculus GN=Mxra7 PE=1 SV=2	MXRA7_MOUSE	19,457.80	99.80%	< 0.001	2	2	2	8.43%
RuvB-like 1 OS=Mus musculus GN=Ruvbl1 PE=1 SV=1	RUVB1_MOUSE	50,215.30	100.00%	< 0.001	11	13	16	31.60%
Selenium-binding protein 1 OS=Mus musculus GN=Selenbp1 PE=1 SV=2	SBP1_MOUSE	52,515.00	100.00%	< 0.001	10	11	13	21.60%
Eukaryotic translation initiation factor 2 subunit 3, Y-linked OS=Mus musculus GN=Eif2s3y PE=2 SV=2	IF2H_MOUSE	51,131.50	100.00%	< 0.001	6	7	7	13.80%
Cleavage and polyadenylation specificity factor subunit 7 OS=Mus musculus GN=Cpsf7 PE=1 SV=2	CPSF7_MOUSE	52,011.40	100.00%	< 0.001	4	4	4	9.98%
SWI/SNF-related matrix-associated actin-dependent regulator of chromatin subfamily E member 1 OS=Mus musculus GN=Smarce1 PE=1 SV=1	SMCE1_MOUSE	46,638.60	100.00%	< 0.001	4	4	4	11.90%
Nucleosome assembly protein 1-like 1 OS=Mus musculus GN=Nap1l1 PE=1 SV=2	NP1L1_MOUSE	45,345.90	100.00%	< 0.001	3	3	3	16.10%
Heterogeneous nuclear ribonucleoprotein H OS=Mus musculus GN=Hnrnph1 PE=1 SV=3	HNRH1_MOUSE	49,199.80	100.00%	< 0.001	2	2	2	5.12%
UBX domain-containing protein 6 OS=Mus musculus GN=Ubxn6 PE=1 SV=1	UBXN6_MOUSE	49,796.90	99.80%	< 0.001	2	2	3	3.39%
Splicing factor 45 OS=Mus musculus GN=Rbm17 PE=1 SV=1	SPF45_MOUSE	45,304.50	99.80%	< 0.001	2	2	2	4.44%
RuvB-like 1 OS=Mus musculus GN=Ruvbl1 PE=1 SV=1	RUVB1_MOUSE	50,215.30	100.00%	< 0.001	31	52	84	54.60%
Ataxin-10 OS=Mus musculus GN=Atxn10 PE=1 SV=2	ATX10_MOUSE	53,708.50	100.00%	< 0.001	25	36	46	47.20%
Cell growth-regulating nucleolar protein OS=Mus musculus GN=Lyar PE=1 SV=2	LYAR_MOUSE	43,735.80	100.00%	< 0.001	20	29	38	42.80%
RecName: Full=Y-box-binding protein 3; AltName: Full=Cold shock	YBOX3_MOUSE	38,813.50	100.00%	< 0.001	18	31	45	40.40%
Nucleosome assembly protein 1-like 4 OS=Mus musculus GN=Nap1l4 PE=1 SV=1	NP1L4_MOUSE	42,679.80	100.00%	< 0.001	16	24	31	42.10%
Splicing factor 45 OS=Mus musculus GN=Rbm17 PE=1 SV=1	SPF45_MOUSE	45,304.50	100.00%	< 0.001	14	16	17	29.60%
Elongation factor 1-alpha 1 OS=Mus musculus GN=Eef1a1 PE=1 SV=3	EF1A1_MOUSE	50,114.20	100.00%	< 0.001	9	10	10	19.90%
Eukaryotic translation initiation factor 2 subunit 3, Y-linked OS=Mus musculus GN=Eif2s3y PE=2 SV=2	IF2H_MOUSE	51,131.50	100.00%	< 0.001	9	10	10	19.90%
Heterogeneous nuclear ribonucleoprotein H OS=Mus musculus GN=Hnrnph1 PE=1 SV=3	HNRH1_MOUSE	49,199.80	100.00%	< 0.001	8	10	13	25.40%
SWI/SNF-related matrix-associated actin-dependent regulator of chromatin subfamily E member 1 OS=Mus musculus GN=Smarce1 PE=1 SV=1	SMCE1_MOUSE	46,638.60	100.00%	< 0.001	7	7	7	20.20%
Y-box-binding protein 2 OS=Mus musculus GN=Ybx2 PE=1 SV=1	YBOX2_MOUSE	38,269.90	100.00%	< 0.001	3	3	4	26.40%
Heterogeneous nuclear ribonucleoprotein K OS=Mus musculus GN=Hnrnpk PE=1 SV=1	HNRPK_MOUSE	50,978.50	100.00%	< 0.001	2	2	2	6.91%
Putative ATP-dependent RNA helicase Pl10 OS=Mus musculus GN=D1Pas1 PE=1 SV=1	DDX3L_MOUSE	73,142.90	100.00%	< 0.001	31	38	54	50.20%
Nuclear RNA export factor 1 OS=Mus musculus GN=Nxf1 PE=1 SV=3	NXF1_MOUSE	70,301.70	100.00%	< 0.001	16	18	21	31.10%
Heterogeneous nuclear ribonucleoprotein Q OS=Mus musculus GN=Syncrip PE=1 SV=2	HNRPQ_MOUSE	69,633.50	100.00%	< 0.001	12	15	15	22.20%
Probable ATP-dependent RNA helicase DDX17 OS=Mus musculus GN=Ddx17 PE=2 SV=1	DDX17_MOUSE	72,401.00	100.00%	< 0.001	11	11	12	20.90%
Tyrosyl-DNA phosphodiesterase 1 OS=Mus musculus GN=Tdp1 PE=2 SV=2	TYDP1_MOUSE	68,691.20	100.00%	< 0.001	10	12	12	22.80%
X-ray repair cross-complementing protein 6 OS=Mus musculus GN=Xrcc6 PE=1 SV=5	XRCC6_MOUSE	69,486.40	100.00%	< 0.001	9	11	11	18.80%
Pre-mRNA 3'-end-processing factor FIP1 OS=Mus musculus GN=Fip1l1 PE=1 SV=1	FIP1_MOUSE	64,959.20	100.00%	< 0.001	7	8	10	18.60%
RNA polymerase II-associated factor 1 homolog OS=Mus musculus GN=Paf1 PE=2 SV=1	PAF1_MOUSE	60,518.60	100.00%	< 0.001	5	5	5	13.80%
Serine/arginine-rich splicing factor 4 OS=Mus musculus GN=Srsf4 PE=2 SV=1	SRSF4_MOUSE	55,981.00	100.00%	< 0.001	4	4	4	6.34%
Probable ATP-dependent RNA helicase DDX4 OS=Mus musculus GN=Ddx4 PE=1 SV=2	DDX4_MOUSE	76,471.40	100.00%	< 0.001	3	5	6	8.40%
Pre-mRNA-splicing factor 38B OS=Mus musculus GN=Prpf38b PE=1 SV=1	PR38B_MOUSE	63,754.10	100.00%	< 0.001	3	4	4	4.06%
E3 ubiquitin/ISG15 ligase TRIM25 OS=Mus musculus GN=Trim25 PE=2 SV=2	TRI25_MOUSE	71,726.00	100.00%	< 0.001	3	3	3	6.78%
Heterochromatin protein 1-binding protein 3 OS=Mus musculus GN=Hp1bp3 PE=1 SV=1	HP1B3_MOUSE	60,868.90	100.00%	< 0.001	2	2	2	4.69%
Ran GTPase-activating protein 1 OS=Mus musculus GN=Rangap1 PE=1 SV=2	RAGP1_MOUSE	63,532.70	99.80%	< 0.001	2	2	2	3.74%
Small ubiquitin-related modifier 1 OS=Mus musculus GN=Sumo1 PE=1 SV=1	SUMO1_MOUSE	11,557.00	99.80%	< 0.001	2	3	3	18.80%
Insulin-like growth factor 2 mRNA-binding protein 3 OS=Mus musculus GN=Igf2bp3 PE=1 SV=1	IF2B3_MOUSE	63,575.60	99.80%	< 0.001	2	2	2	5.18%
Switch-associated protein 70 OS=Mus musculus GN=Swap70 PE=1 SV=2	SWP70_MOUSE	68,997.90	99.80%	< 0.001	2	2	2	3.42%
Splicing factor, proline- and glutamine-rich OS=Mus musculus GN=Sfpq PE=1 SV=1	SFPQ_MOUSE	75,441.90	100.00%	< 0.001	33	54	65	37.90%
Far upstream element-binding protein 2 OS=Mus musculus GN=Khsrp PE=1 SV=2	FUBP2_MOUSE	76,775.20	100.00%	< 0.001	27	41	71	40.80%
Putative pre-mRNA-splicing factor ATP-dependent RNA helicase DHX15 OS=Mus musculus GN=Dhx15 PE=2 SV=2	DHX15_MOUSE	91,009.30	100.00%	< 0.001	22	30	40	27.90%
ATP-dependent RNA helicase DDX1 OS=Mus musculus GN=Ddx1 PE=1 SV=1	DDX1_MOUSE	82,501.10	100.00%	< 0.001	17	21	24	25.80%
DNA replication licensing factor MCM5 OS=Mus musculus GN=Mcm5 PE=2 SV=1	MCM5_MOUSE	82,345.70	100.00%	< 0.001	10	10	11	12.70%
