# Supplementary material for: Senataxin controls meiotic silencing through ATR activation and chromatin remodeling
Source: Cell Discov. 2015 Sep 29;1:15025–. doi: 10.1038/celldisc.2015.25 (PMC4860845; doi:10.1038/celldisc.2015.25)
Supplement: Supplementary Table S2 [file celldisc201525-s7.pdf]

Supplementary Table 2. Primers used in gene expression analysis and R-loop detection

ATRIP 1F	5’ GGAACCTGTCCAGTGCTCAT 3’
ATRIP 1R	5’ AGGATTTCTTCTTCCATTGCTTTC 3’
TOPBP1 1F	5’ TGTGTGACCCTGCAATGGTT 3’
TOPBP1 1R	5’ ACACAGCTGCCATTTATGTTG 3’
NEK1 IF	5’ AAATGCTCAGAAAGGCGCTC 3’
NEK1 1R	5’ ATCTCCAAGCTGCACTGTCC 3’
GAPDH 1F	5’ ATTGTCAGCAATGCATCCTG 3’
GAPDH 1R	5’ ATTGTCAGCAATGCATCCTG 3’
TIMP1 RLFS 1F	5’ GCTGGCTGGCAGAGGAG 3’
TIMP1 RLFS 1R	5’ GGCTTCCTGGACGCATTACT 3’
